# Supplementary material for: Long-term balancing selection for pathogen resistance maintains trans-species polymorphisms in a planktonic crustacean
Source: Nat Commun. 2024 Jun 22;15:5333. doi: 10.1038/s41467-024-49726-8 (PMC11193740; doi:10.1038/s41467-024-49726-8)
Supplement: Supplementary file 1 — Supplementary Information [file 41467_2024_49726_MOESM1_ESM.pdf]

Supplementary Figures and Tables for

Long-term balancing selection for pathogen resistance maintains trans-species polymorphisms in a planktonic crustacean

Luca Cornetti[1,+], Peter D. Fields[1], Louis Du Pasquier[1] & Dieter Ebert[1] ✉

Nature communications (2024)

Affiliations:

[1] *University of Basel, Department of Environmental Sciences, Zoology, Basel, Switzerland*

✉ email: dieter.ebert@unibas.ch

+ current address: Syngenta Crop Protection AG, CH-4332 Stein, Switzerland

orcID:

Luca Cornetti	0000-0002-7188-4048
Peter D. Fields	0000-0003-2959-2524
Louis Du Pasquier	0000-0003-2930-8968
Dieter Ebert	0000-0003-2653-3772

Supplementary Figure 1. Principal Component Analysis (PCA) based on 92608 unlinked SNPs describing species diversification including *D. magna* (blue), *D. similis* (yellow) and *D. sinensis* (green). Since PCA are sensitive to sample size, we subsampled our large *D. magna* sample to include only 15 randomly chosen genotypes. Each plot in this panel of 9 shows a different subsample. The samples sizes for *D. similis* and *D.* sinensis were 15 and 14, respectively. The panels confirm the structure of the entire dataset (Fig. 1b). Source data are provided as a Source Data file.

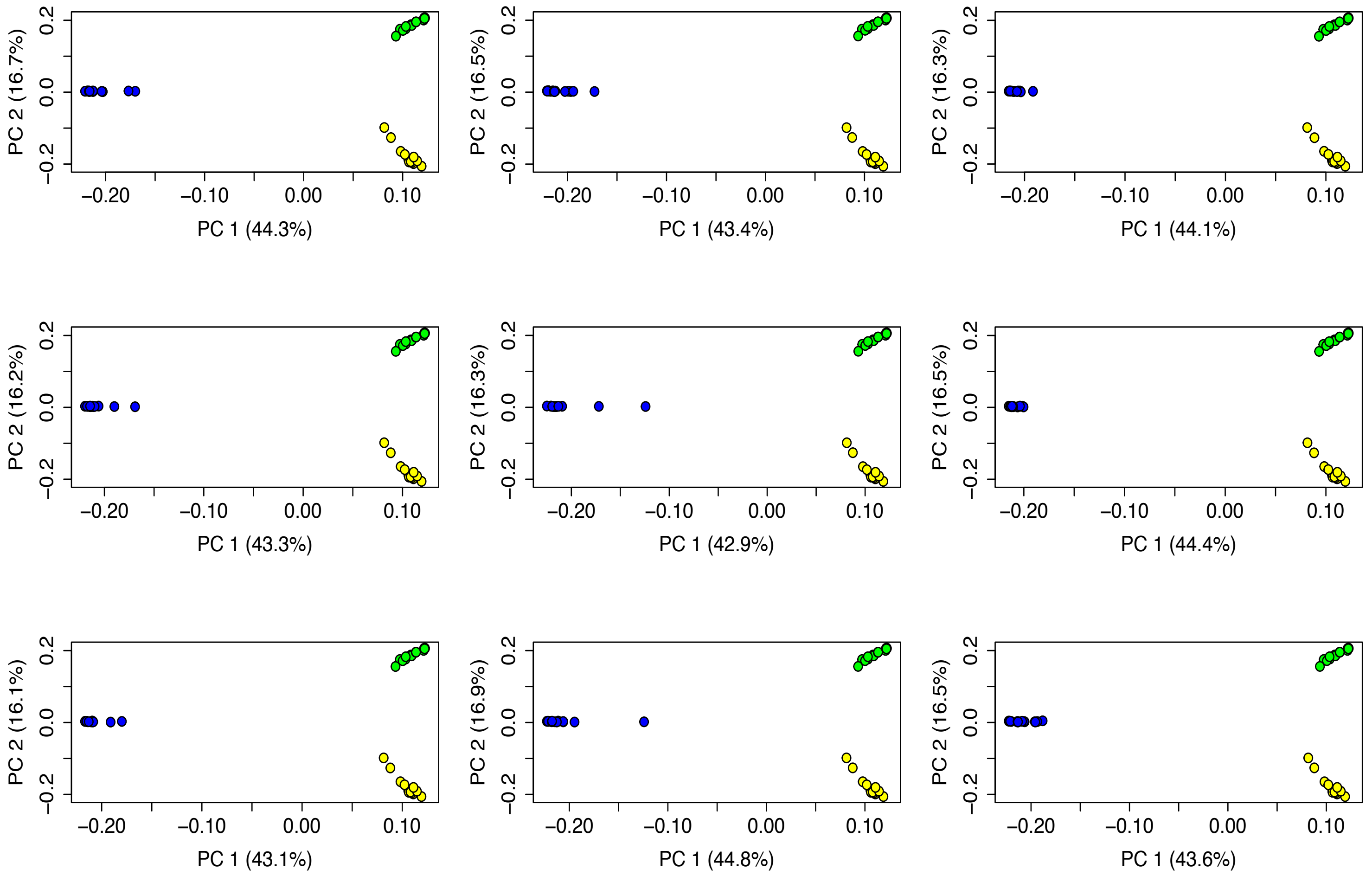

Supplementary Figure 2. Principal Component Analysis (PCA) based on 92608 unlinked SNPs describing species diversification including *D. magna* and *D. similis* genotypes. *D. magna* WE is the Western Eurasian clade of this species, EA is the East Asian clade and NA the North American clade. Source data are provided as a Source Data file.

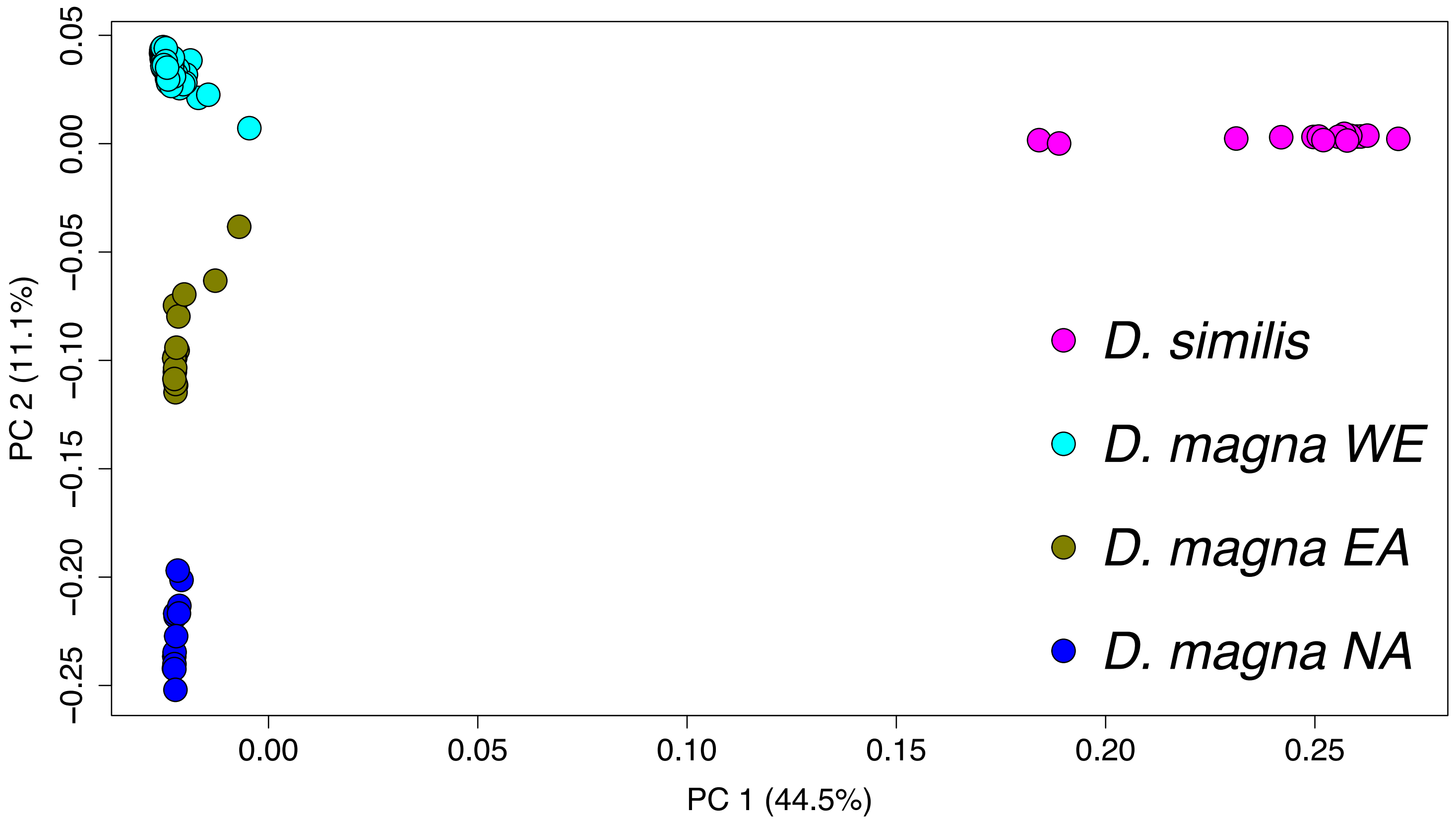

Supplementary Figure 3. Principal Component Analysis (PCA) based on 92608 unlinked SNPs describing species diversification including *D. magna* and *D. sinensis* genotypes. *D. magna* WE is the Western Eurasian clade of this species, EA is the East Asian clade and NA the North American clade. Source data are provided as a Source Data file.

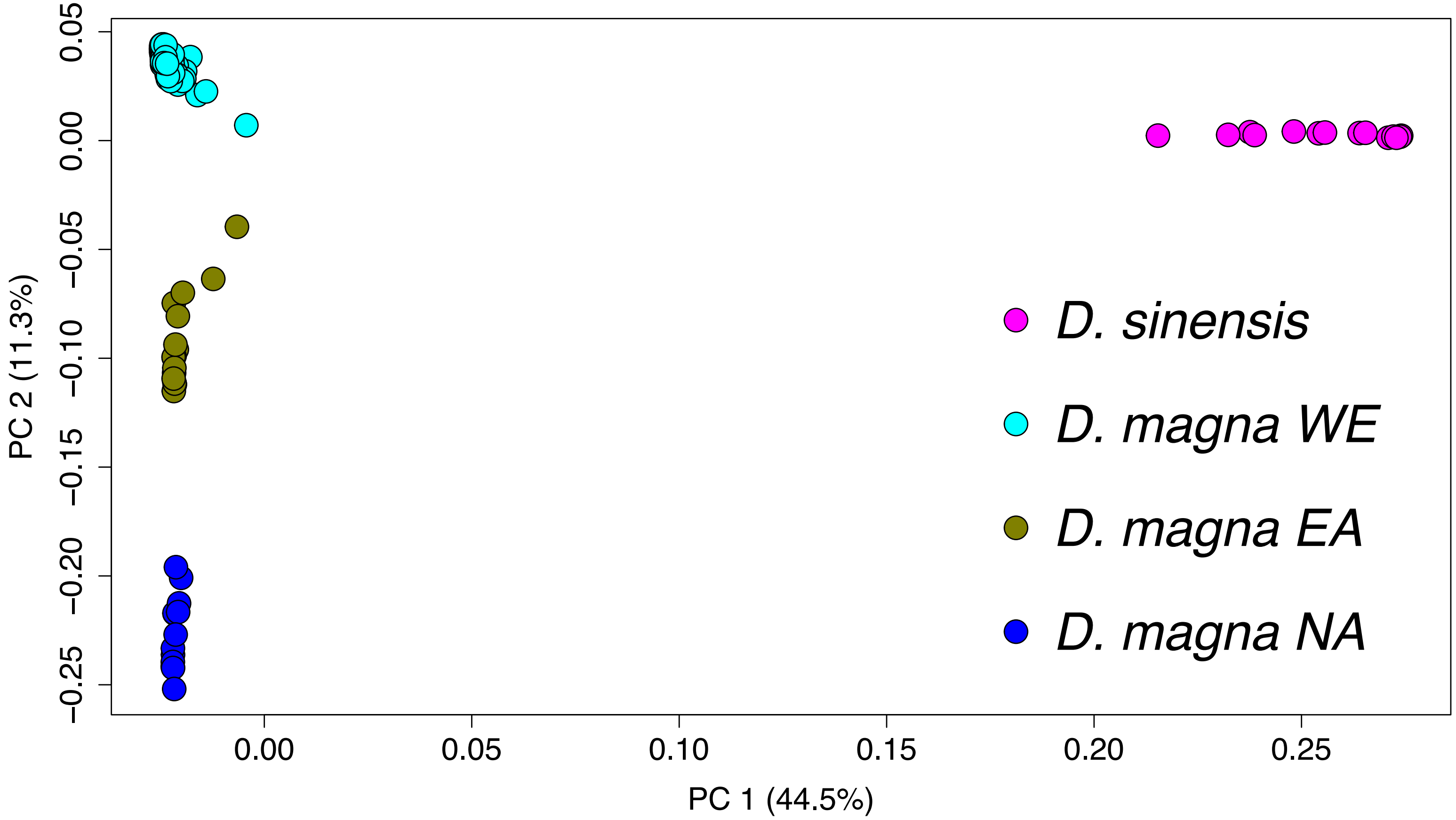

Supplementary Figure 4. Principal Component Analysis (PCA) based on 92608 unlinked SNPs describing species diversification including *D. similis* and *D. sinensis* genotypes. Source data are provided as a Source Data file.

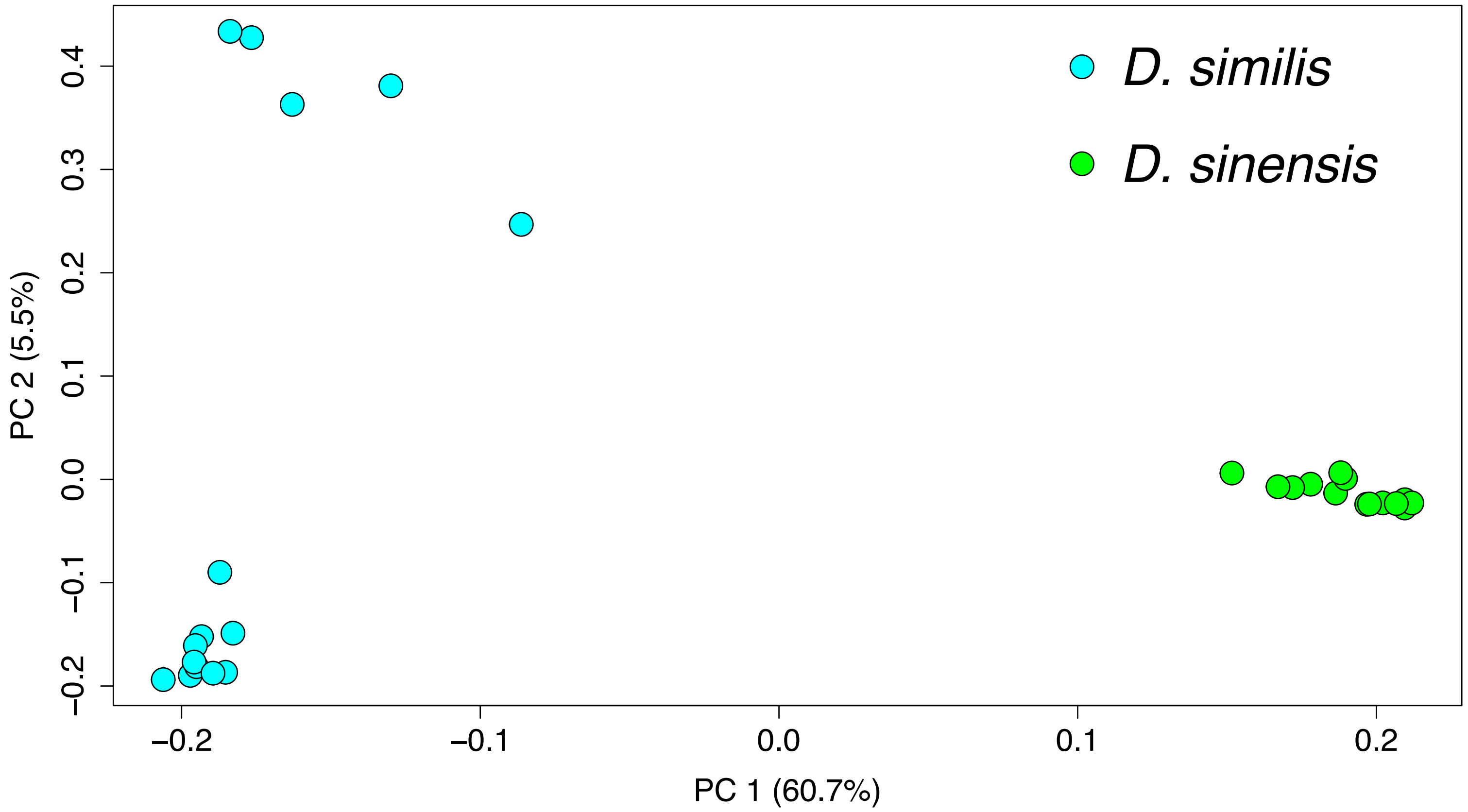

Supplementary Figure 5. Principal Component Analysis (PCA) based on 92608 unlinked SNPs describing species diversification including all *D. magna* genotypes. *D. magna* WE is the Western Eurasian clade of this species, EA is the East Asian clade and NA the North American clade. Source data are provided as a Source Data file.

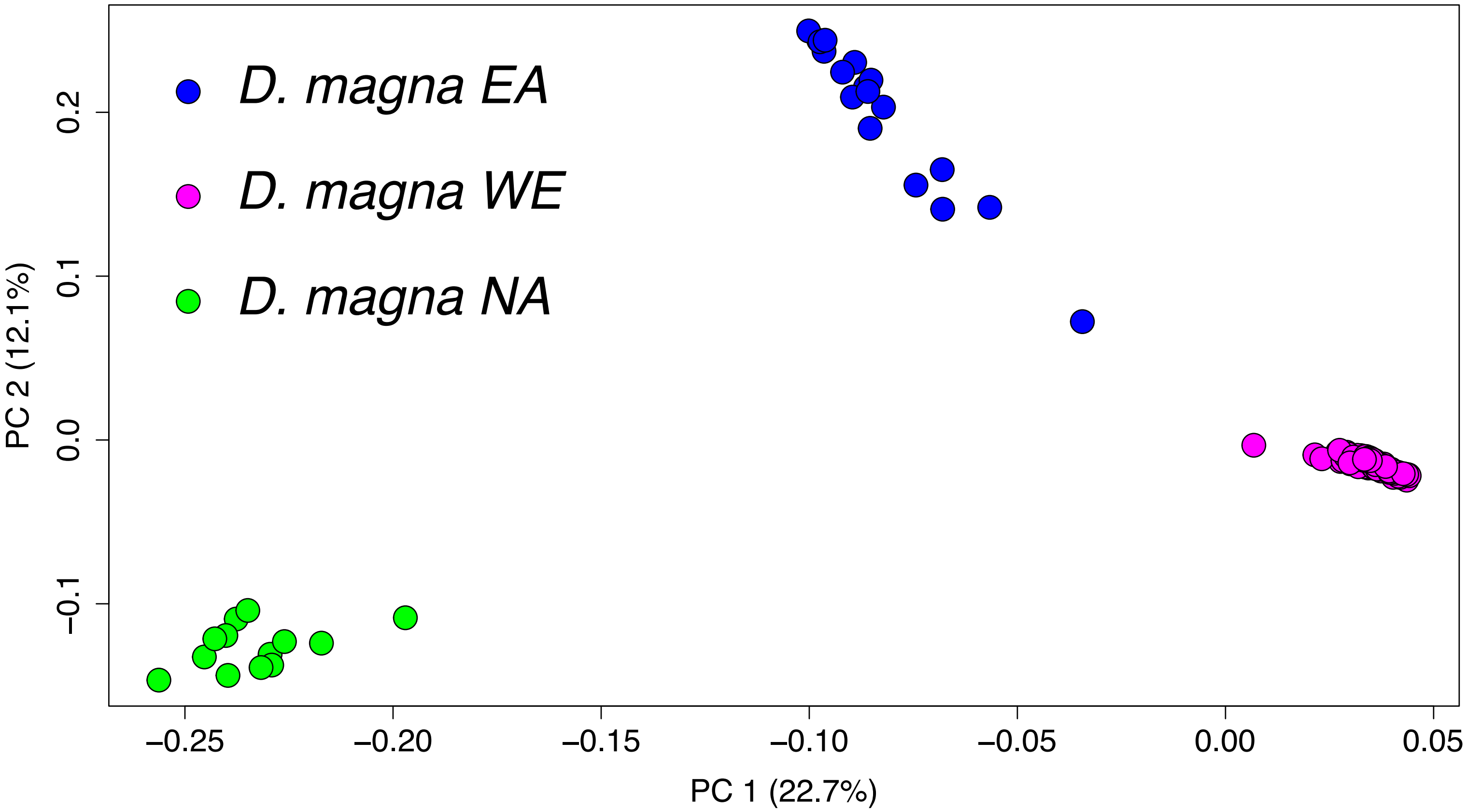

Supplementary Figure 6. Best ML mitochondrial phylogeny including a subset of the Daphnia genotypes analyzed in this study. *D. magna* WE is the Western Eurasian clade of this species, EA is the East Asian clade and NA the North American clade. *D. hispanica* was used as an outgroup, as it is closely related to the other species. Source data are provided as a Source Data file.

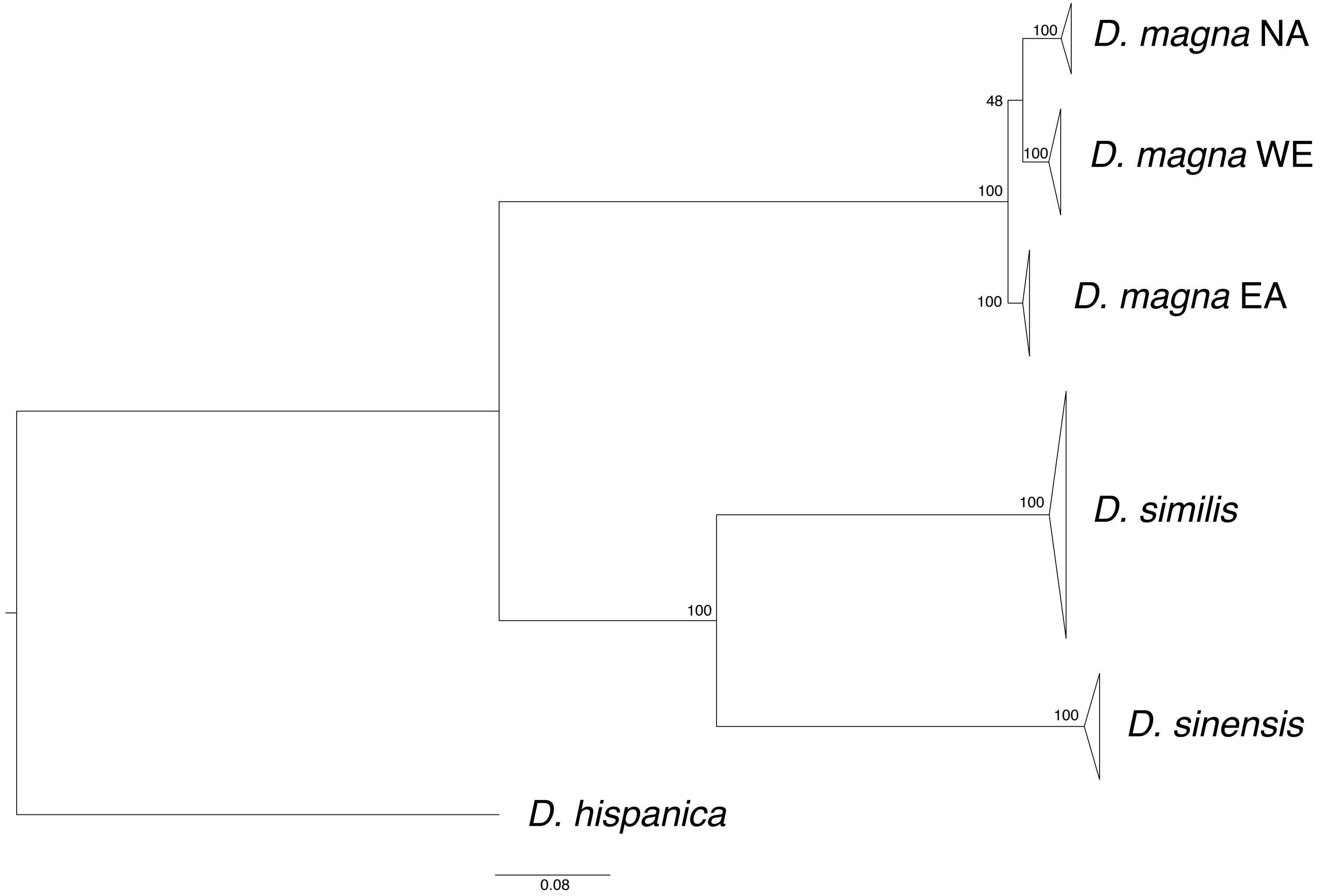

Supplementary Figure 7. Dot plots (left) of nucleotide diversity (π) along contig 11F, where two putative TSPs were identified. Each dot is the average of π in a 5kb non-overlapping sliding window. The red line represents the moving average of π along the contig, while the horizontal dotted line the overall average of π in the contig. The blue and brown dashed vertical lines indicate the coordinates of the ABC locus and the F locus, respectively. The green triangles indicate the position of the two TSPs. Violin plots (right) displaying the distribution of π in the candidate regions and in the background. The P-values (two-sided Wilcoxon tests) associated with the statistical difference between the different groups are reported. *Daphnia magna* plots are showed at the top, *D. similis* plots in the middle and *D. sinensis* plots at the bottom of the figure. CR_1: candidate region in the surrounding of the ABC locus; BG_1: background of contig 11F relative to CR_1; CR_2: candidate region within the F locus; BG_2: background of contig 11F relative to CR_2; 20 contigs: π calculated as background in the 20 longest contigs. Source data are provided as a Source Data file.

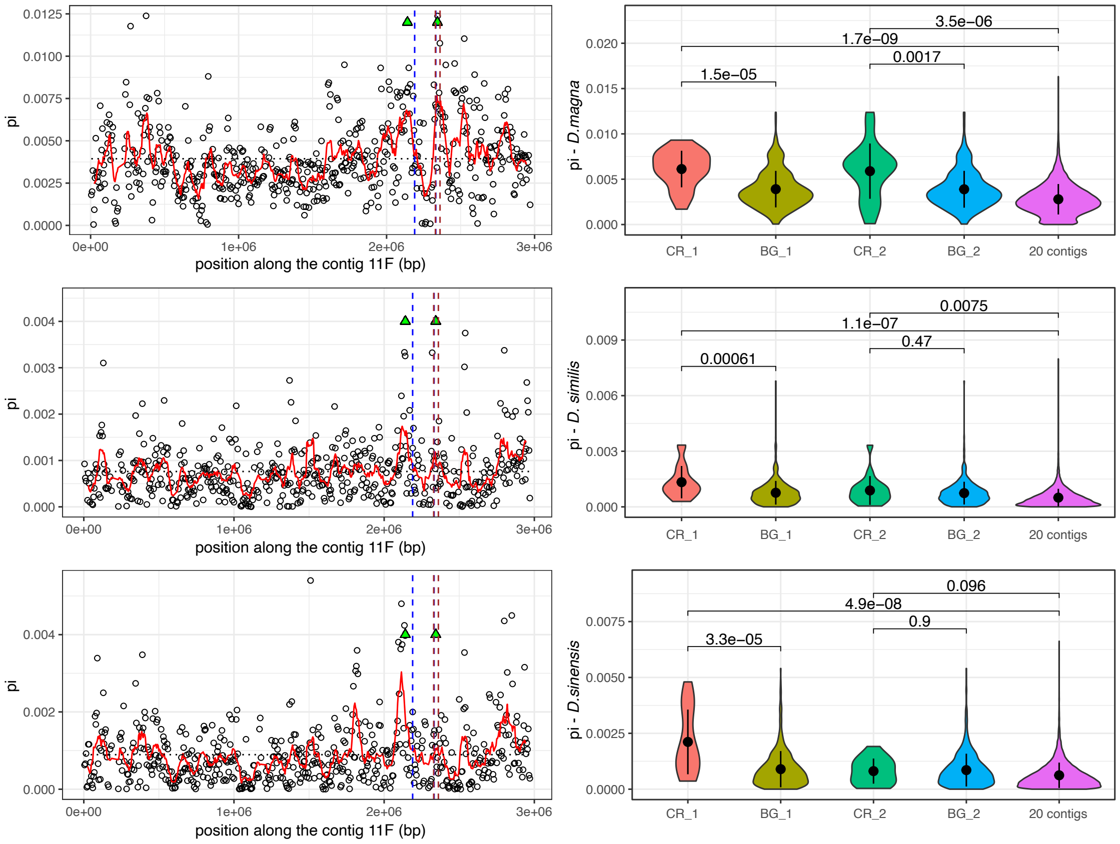

Supplementary Figure 8. Dot plots (left) of nucleotide diversity (π) along contig 18F, where one putative TSP was identified. Each dot is the average of π in a 5kb non-overlapping sliding window. The red line represents the moving average of π along the contig, while the horizontal dotted line the overall average of π in the contig. The dashed vertical lines indicate the coordinates of the D locus. The green triangle indicates the position of the TSP. Violin plots (right) displaying the distribution of π in the candidate region and in the background. The P-values (two-sided Wilcoxon tests) associated with the statistical difference between the different groups are reported. *Daphnia magna* plots are showed at the top, *D. similis* plots in the middle and *D. sinensis* plots at the bottom of the figure. CR: candidate region; BG: background of contig 18F; 20 contigs: π calculated as background in the 20 longest contigs. Source Data are provided as a Source Data file.

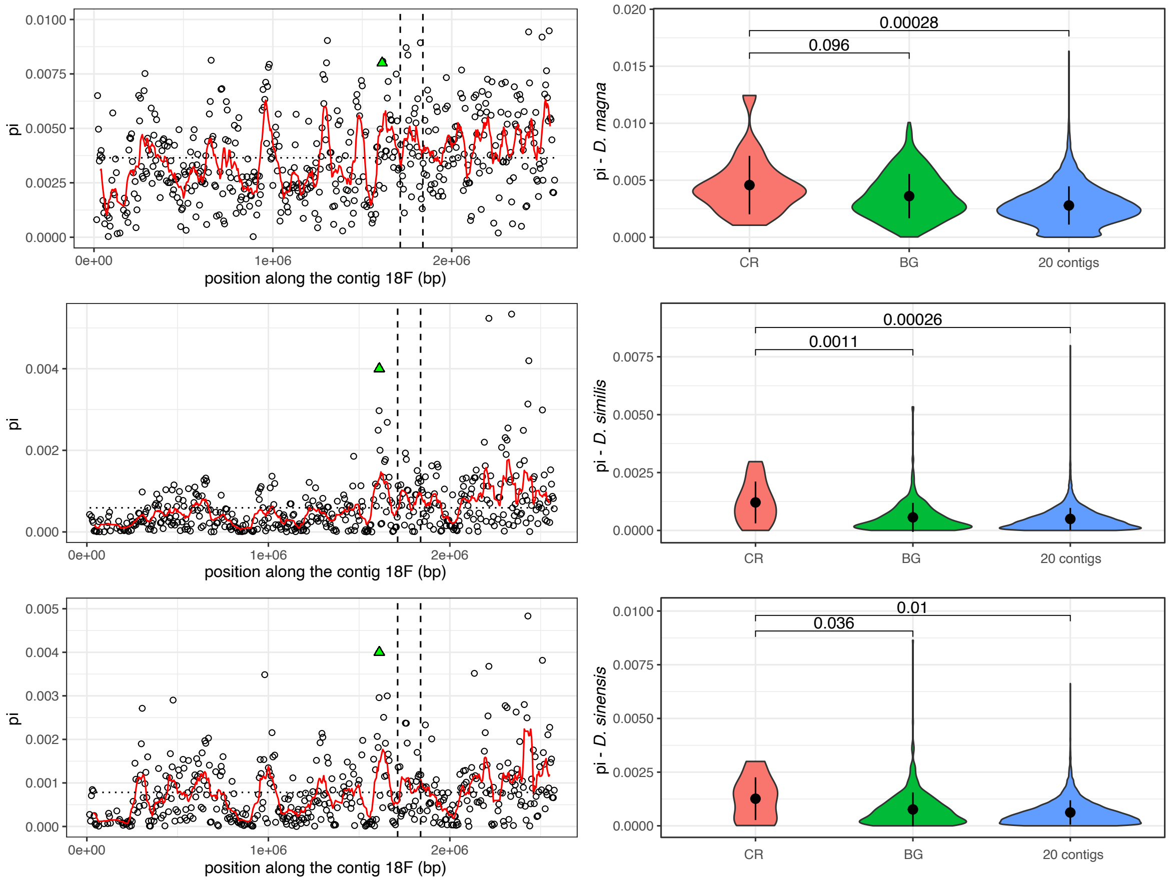

Supplementary Figure 9. Dot plots (left) of nucleotide diversity (π) along contig 28F, where two putative TSPs were identified. Each dot is the average of π in a 5kb non-overlapping sliding window. The red line represents the moving average of π along the contig, while the horizontal dotted line the overall average of π in the contig. The green triangles indicate the position of the TSPs. Violin plots (right) displaying the distribution of π in the candidate region and in the background. The P-values (two-sided Wilcoxon tests) associated with the statistical difference between the different groups are reported. *Daphnia magna* plots are showed at the top, *D. similis* plots in the middle and *D. sinensis* plots at the bottom of the figure. CR: candidate region; BG: background of contig 28F; 20 contigs: π calculated as background in the 20 longest contigs. Source data are provided as a Source Data file.

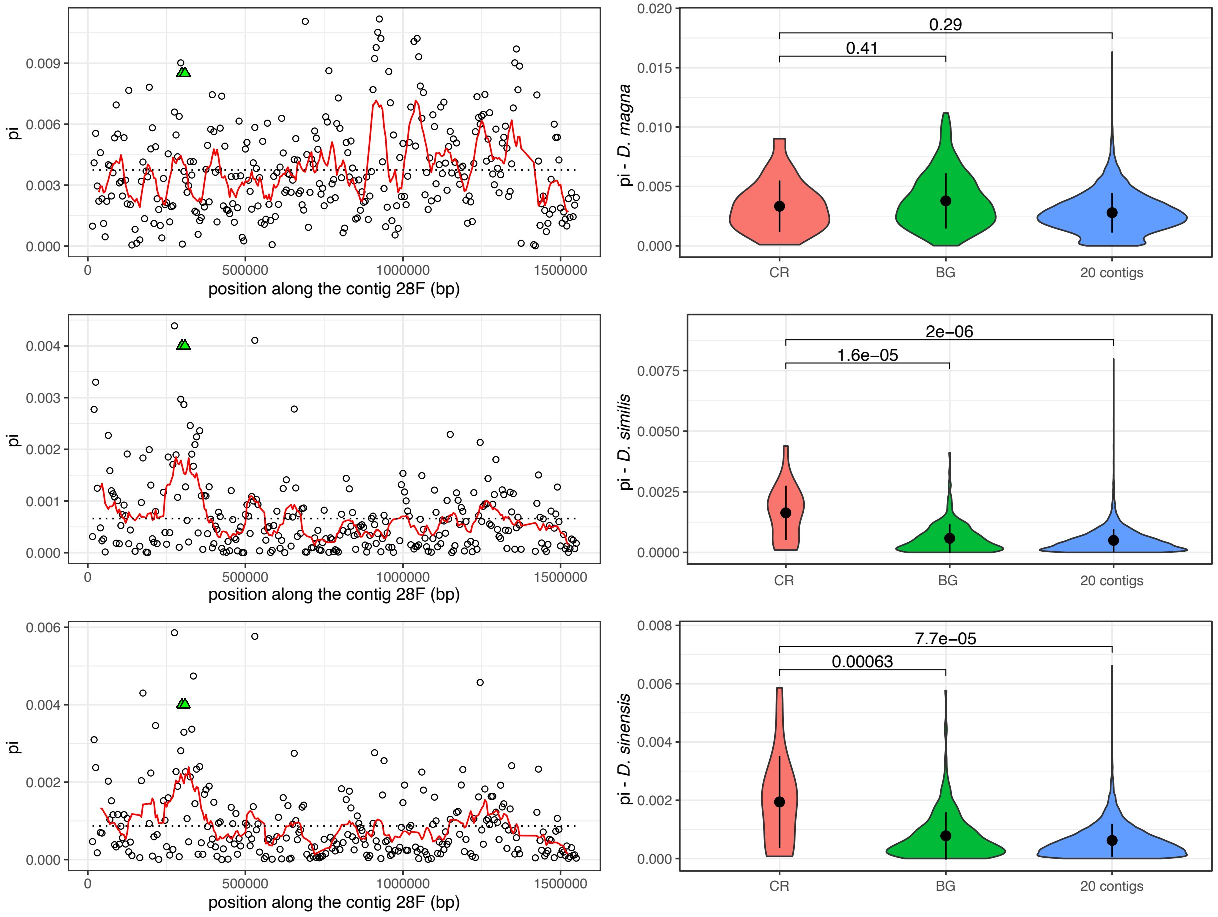

Supplementary Figure 10. Dot plots (left) of Tajima's D along contig 11F, where two putative TSPs were identified. Each dot is the average of Tajima's D in a 5kb non-overlapping sliding window. The red line represents the moving average of Tajima's D along the contig, while the horizontal dotted line the overall average of Tajima's D in the contig. The blue and brown dashed vertical lines indicate the coordinates of the ABC locus and the F locus, respectively. The green triangles indicate the position of the two TSPs. Violin plots (right) displaying the distribution of Tajima's D in the candidate regions and in the background. The P-values (two-sided Wilcoxon tests) associated with the statistical difference between the different groups are reported. *Daphnia magna* plots are showed at the top, *D. similis* plots in the middle and *D. sinensis* plots at the bottom of the figure. CR_1: candidate region in the surrounding of the ABC locus; BG_1: background of contig 11F relative to CR_1; CR_2: candidate region within the F locus; BG_2: background of contig 11F relative to CR_2; 20 contigs: Tajima's D calculated as background in the 20 longest contigs. Source data are provided as a Source Data file.

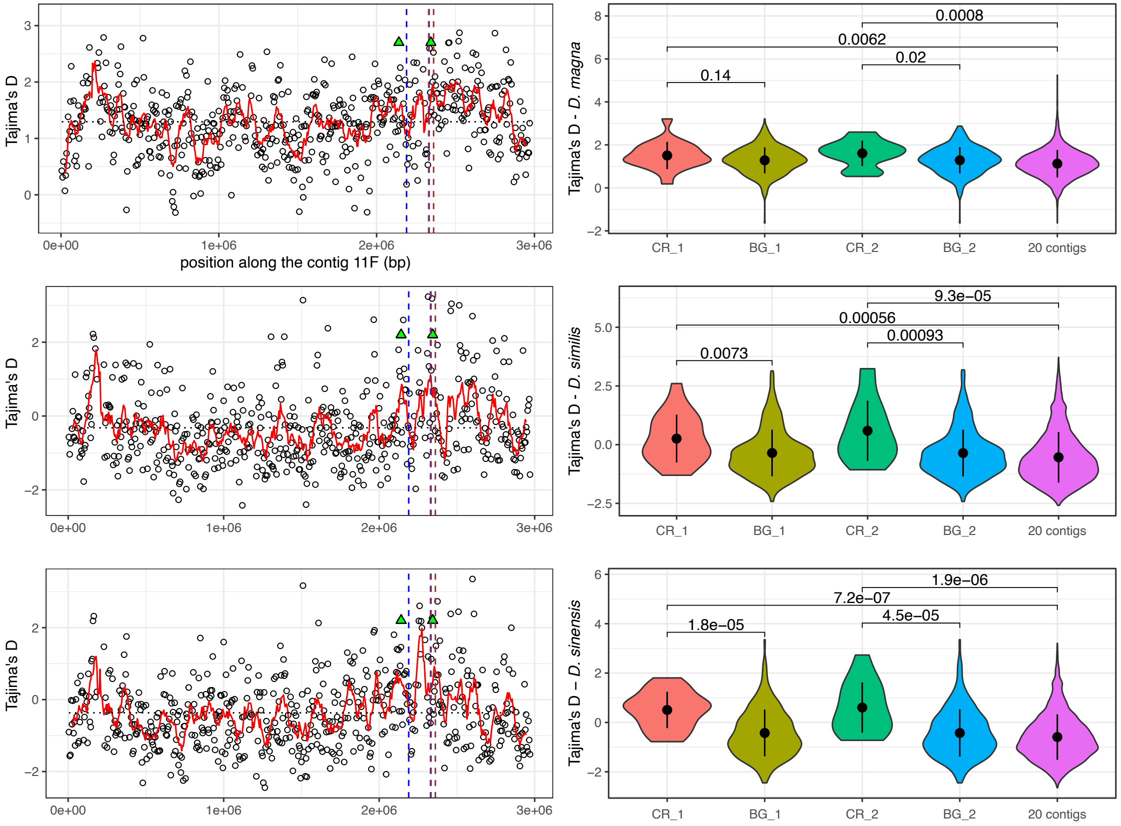

Supplementary Figure 11. Dot plots (left) of Tajima's D along contig 18F, where one putative TSP was identified. Each dot is the average of Tajima's D in a 5kb non-overlapping sliding window. The red line represents the moving average of Tajima's D along the contig, while the horizontal dotted line the overall average of Tajima's D in the contig. The dashed vertical lines indicate the coordinates of the D locus. The green triangle indicates the position of the TSP. Violin plots (right) displaying the distribution of Tajima's D in the candidate region and in the background. The P-values (two-sided Wilcoxon tests) associated with the statistical difference between the different groups are reported. *Daphnia magna* plots are showed at the top, *D. similis* plots in the middle and *D. sinensis* plots at the bottom of the figure. CR: candidate region; BG: background of contig 18F; 20 contigs: Tajima's D calculated as background in the 20 longest contigs. Source data are provided as a Source Data file.

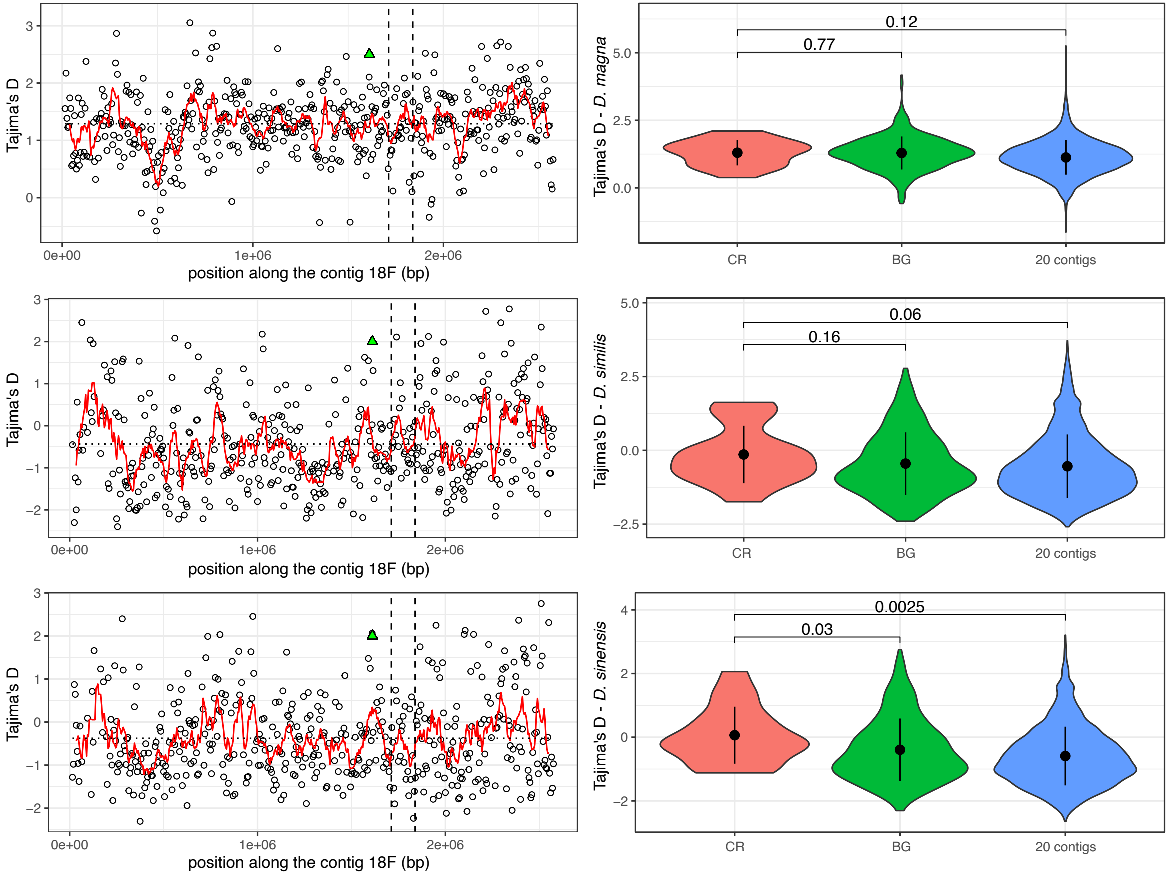

Supplementary Figure 12. Dot plots (left) of Tajima's D along contig 28F, where two putative TSPs were identified (only 1 remained in the final set of candidates). Each dot is the average of Tajima's D in a 5kb non-overlapping sliding window. The red line represents the moving average of Tajima's D along the contig, while the horizontal dotted line the overall average of Tajima's D in the contig. The green triangles indicate the position of the TSPs. Violin plots (right) displaying the distribution of Tajima's D in the candidate region and in the background. The P-values (two-sided Wilcoxon tests) associated with the statistical difference between the different groups are reported. *Daphnia magna* plots are showed at the top, *D. similis* plots in the middle and *D. sinensis* plots at the bottom of the figure. CR: candidate region; BG: background of contig 28F; 20 contigs: Tajima's D calculated as background in the 20 longest contigs. Source data are provided as a Source Data file.

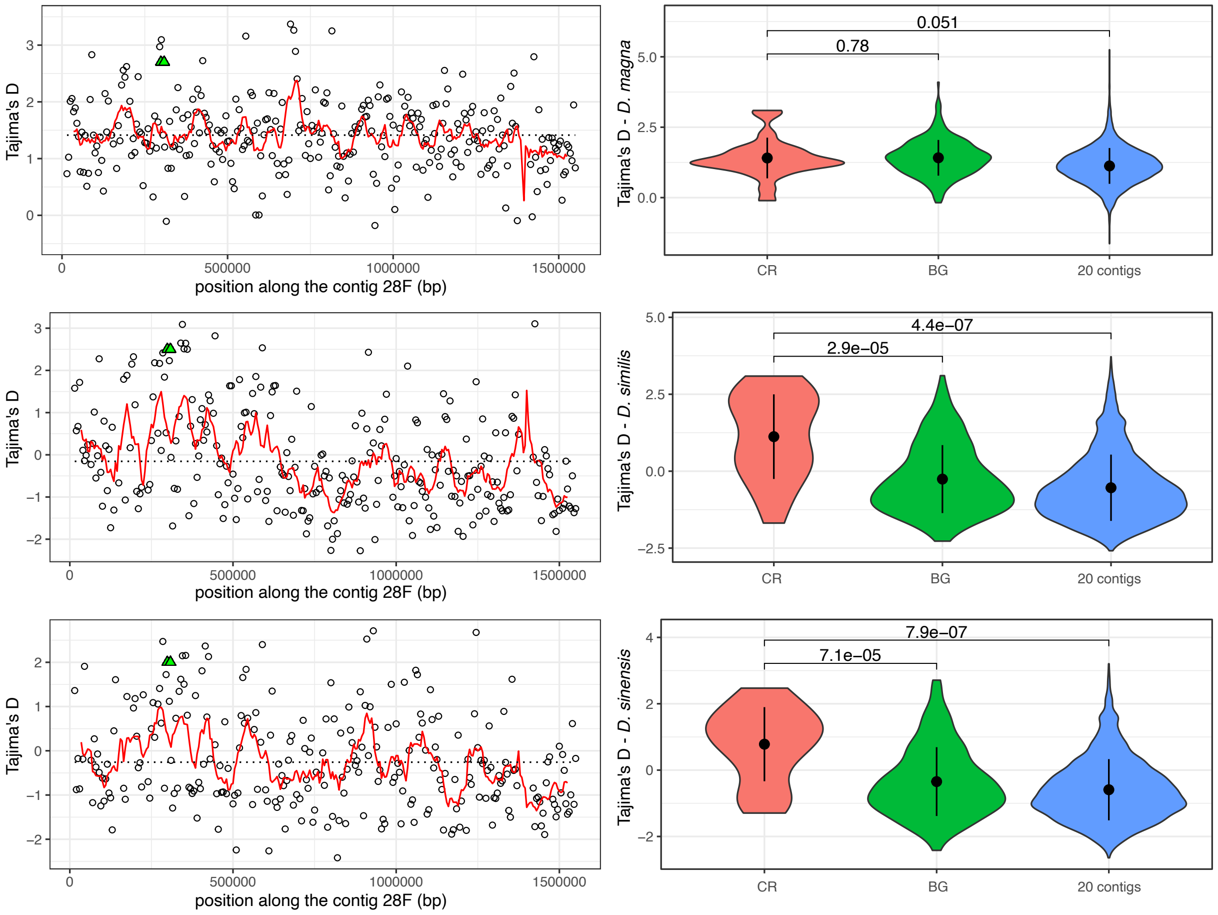

Supplementary Figure 13. Dot plots (left) of F_{st} along contig 11F, where two putative TSPs were identified. Each dot is the average of F_{st} in a 5kb non-overlapping sliding window. The red line represents the moving average of F_{st} along the contig, while the horizontal dotted line the overall average of F_{st} in the contig. The blue and brown dashed vertical lines indicate the coordinates of the ABC locus and the F locus, respectively. The green triangles indicate the position of the two TSPs. Violin plots (right) displaying the distribution of F_{st} in the candidate regions and in the background. The P-values (two-sided Wilcoxon tests) associated with the statistical difference between the different groups are reported. F_{st} between *D. magna* and *D. similis* plots are showed at the top, F_{st} between *D. similis* and *D. sinensis* plots in the middle and F_{st} between *D. magna* and *D. sinensis* plots at the bottom of the figure. CR_1: candidate region in the surrounding of the ABC locus; BG_1: background of contig 11F relative to CR_1; CR_2: candidate region within the F locus; BG_2: background of contig 11F relative to CR_2; 20 contigs: F_{st} calculated as background in the 20 longest contigs. Source data are provided as a Source Data file.

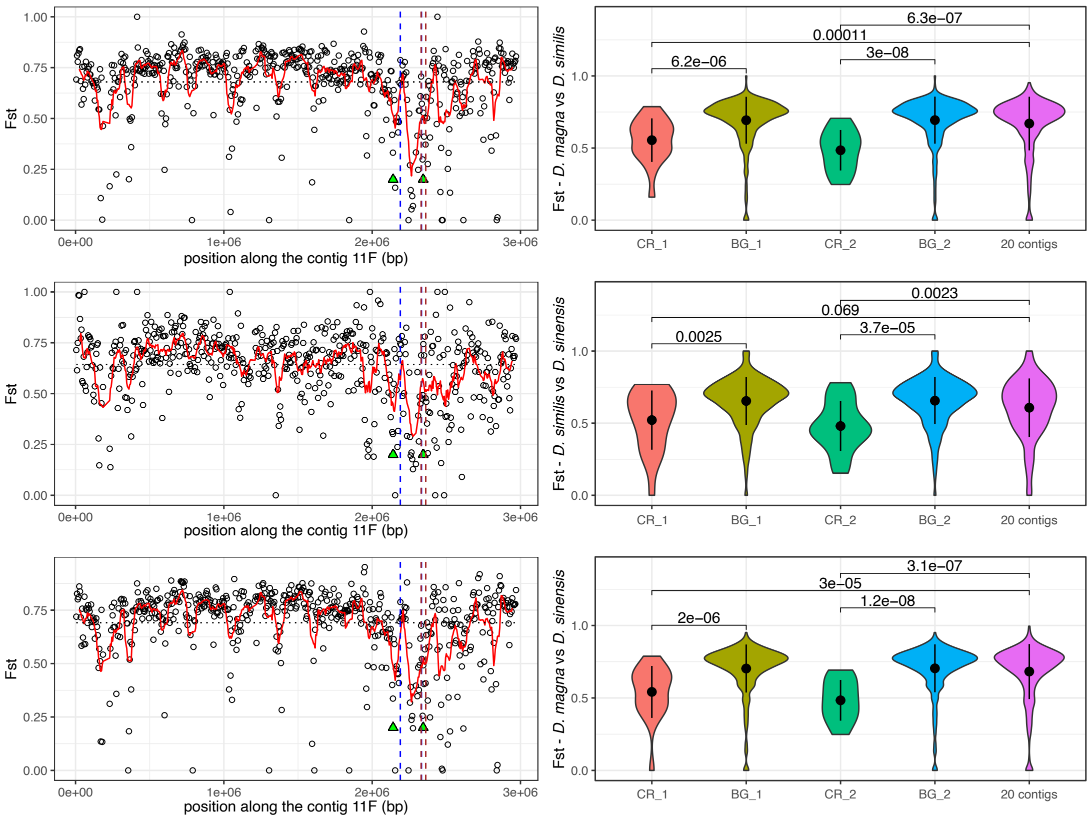

Supplementary Figure 14. Dot plots (left) of F_{st} along contig 18F, where one putative TSP was identified. Each dot is the average of F_{st} in a 5kb non-overlapping sliding window. The red line represents the moving average of F_{st} along the contig, while the horizontal dotted line the overall average of F_{st} in the contig. The dashed vertical lines indicate the coordinates of the D locus. The green triangle indicates the position of the TSP. Violin plots (right) displaying the distribution of F_{st} in the candidate region and in the background. The P-values (two-sided Wilcoxon tests) associated with the statistical difference between the different groups are reported. F_{st} between *D. magna* and *D. similis* plots are showed at the top, F_{st} between *D. similis* and *D. sinensis* plots in the middle and F_{st} between *D. magna* and *D. sinensis* plots at the bottom of the figure. CR: candidate region; BG: background of contig 18F; 20 contigs: F_{st} calculated as background in the 20 longest contigs. Source data are provided as a Source Data file.

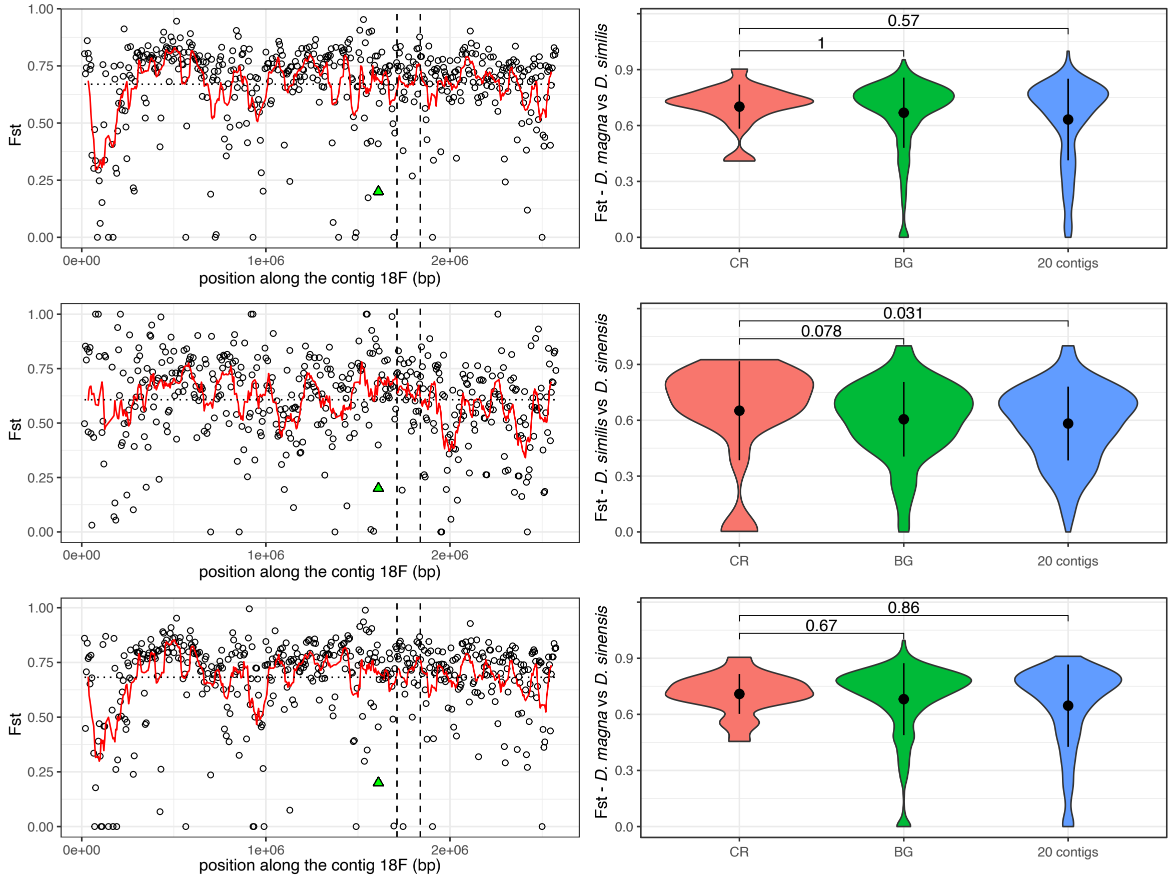

Supplementary Figure 15. Dot plots (left) of F_{st} along contig 28F, where two putative TSPs were identified (only 1 remained in the final set of candidates). Each dot is the average of F_{st} in a 5kb non-overlapping sliding window. The red line represents the moving average of F_{st} along the contig, while the horizontal dotted line the overall average of F_{st} in the contig. The green triangles indicate the position of the TSP. Violin plots (right) displaying the distribution of F_{st} in the candidate region and in the background. The P-values (two-sided Wilcoxon tests) associated with the statistical difference between the different groups are reported. F_{st} between *D. magna* and *D. similis* plots are showed at the top, F_{st} between *D. similis* and *D. sinensis* plots in the middle and F_{st} between *D. magna* and *D. sinensis* plots at the bottom of the figure. CR: candidate region; BG: background of contig 28F; 20 contigs: F_{st} calculated as background in the 20 longest contigs. Source data are provided as a Source Data file.

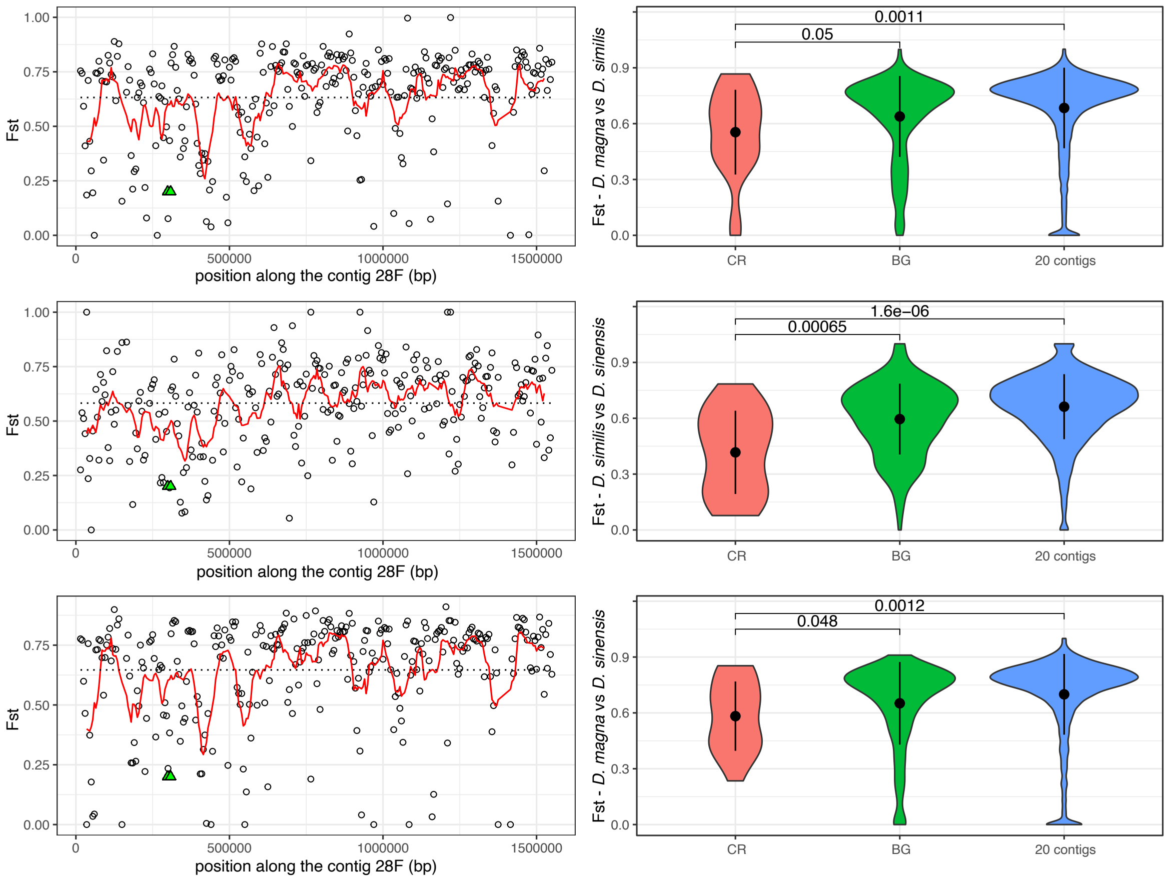

Supplementary Figure 16. Dot plots (left) of beta scores (using -maf 0.05) along contig 11F, where two putative TSPs were identified. Each dot is the average of beta in a 5kb non-overlapping sliding window. The red line represents the moving average of beta along the contig, while the horizontal dotted line the overall average of beta in the contig. The blue and brown dashed vertical lines indicate the coordinates of the ABC locus and the F locus, respectively. The green triangles indicate the position of the two TSPs. Violin plots (right) displaying the distribution of beta in the candidate regions and in the background. The P-values (two-sided Wilcoxon tests) associated with the statistical difference between the different groups are reported. *Daphnia magna* plots are showed at the top, *D. similis* plots in the middle and *D. sinensis* plots at the bottom of the figure. CR_1: candidate region in the surrounding of the ABC locus; BG_1: background of contig 11F relative to CR_1; CR_2: candidate region within the F locus; BG_2: background of contig 11F relative to CR_2; 20 contigs: beta calculated as background in the 20 longest contigs. Source data are provided as a Source Data file.

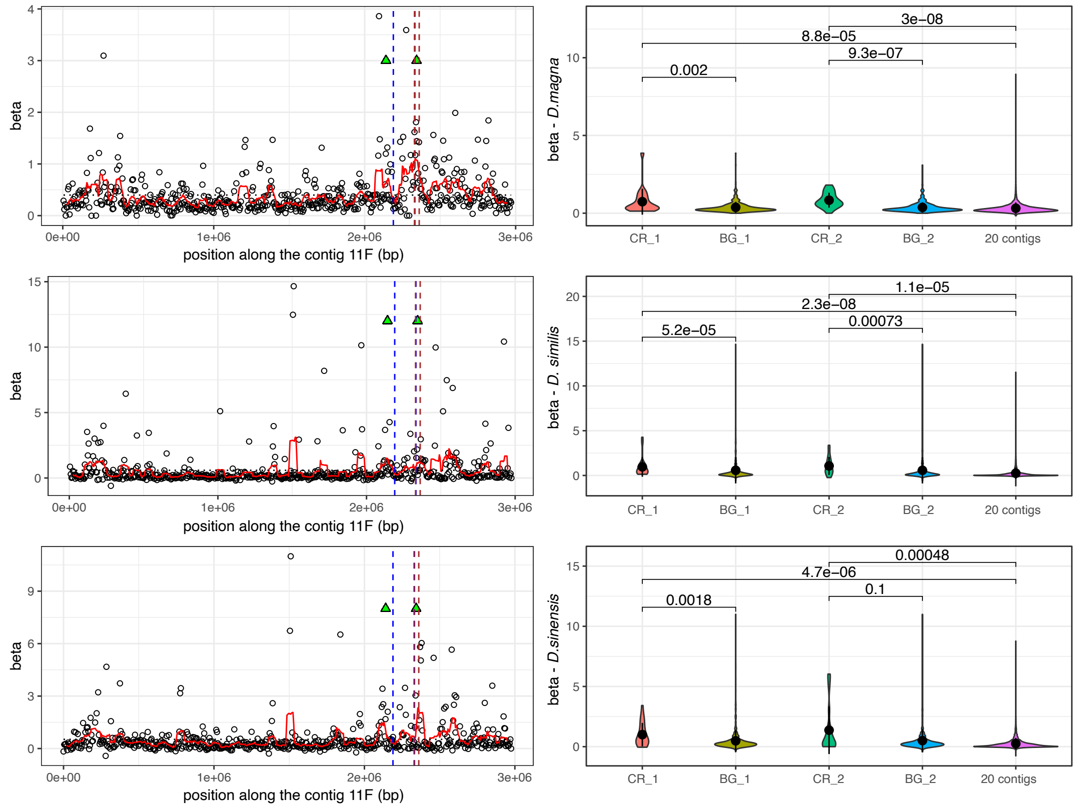

Supplementary Figure 17. Dot plots (left) of beta scores (using -maf 0.05) along contig 18F, where one putative TSP was identified. Each dot is the average of beta in a 5kb non-overlapping sliding window. The red line represents the moving average of beta along the contig, while the horizontal dotted line the overall average of beta in the contig. The dashed vertical lines indicate the coordinates of the D locus. The green triangle indicates the position of the TSP. Violin plots (right) displaying the distribution of beta in the candidate region and in the background. The P-values (two-sided Wilcoxon tests) associated with the statistical difference between the different groups are reported. *Daphnia magna* plots are showed at the top, *D. similis* plots in the middle and *D. sinensis* plots at the bottom of the figure. CR: candidate region; BG: background of contig 18F; 20 contigs: beta calculated as background in the 20 longest contigs. Source data are provided as a Source Data file.

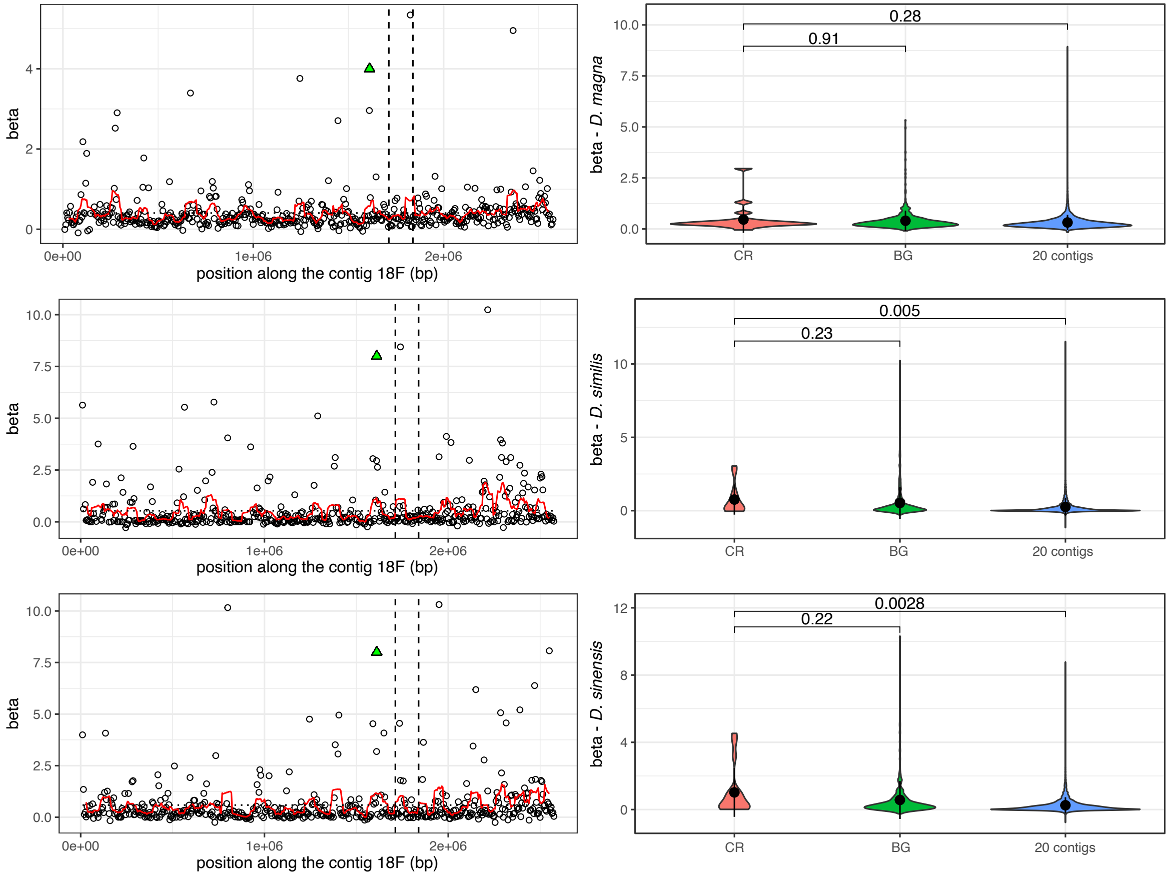

Supplementary Figure 18. Dot plots (left) of beta scores (using -maf 0.05) along contig 28F, where two putative TSPs were identified (only 1 remained in the fianl set of candidates). Each dot is the average of beta in a 5kb non-overlapping sliding window. The red line represents the moving average of beta along the contig, while the horizontal dotted line the overall average of beta in the contig. The green triangles indicate the position of the TSPs. Violin plots (right) displaying the distribution of beta in the candidate region and in the background. The P-values (two-sided Wilcoxon tests) associated with the statistical difference between the different groups are reported. *Daphnia magna* plots are showed at the top, *D. similis* plots in the middle and *D. sinensis* plots at the bottom of the figure. CR: candidate region; BG: background of contig 28F; 20 contigs: beta calculated as background in the 20 longest contigs. Source data are provided as a Source Data file.

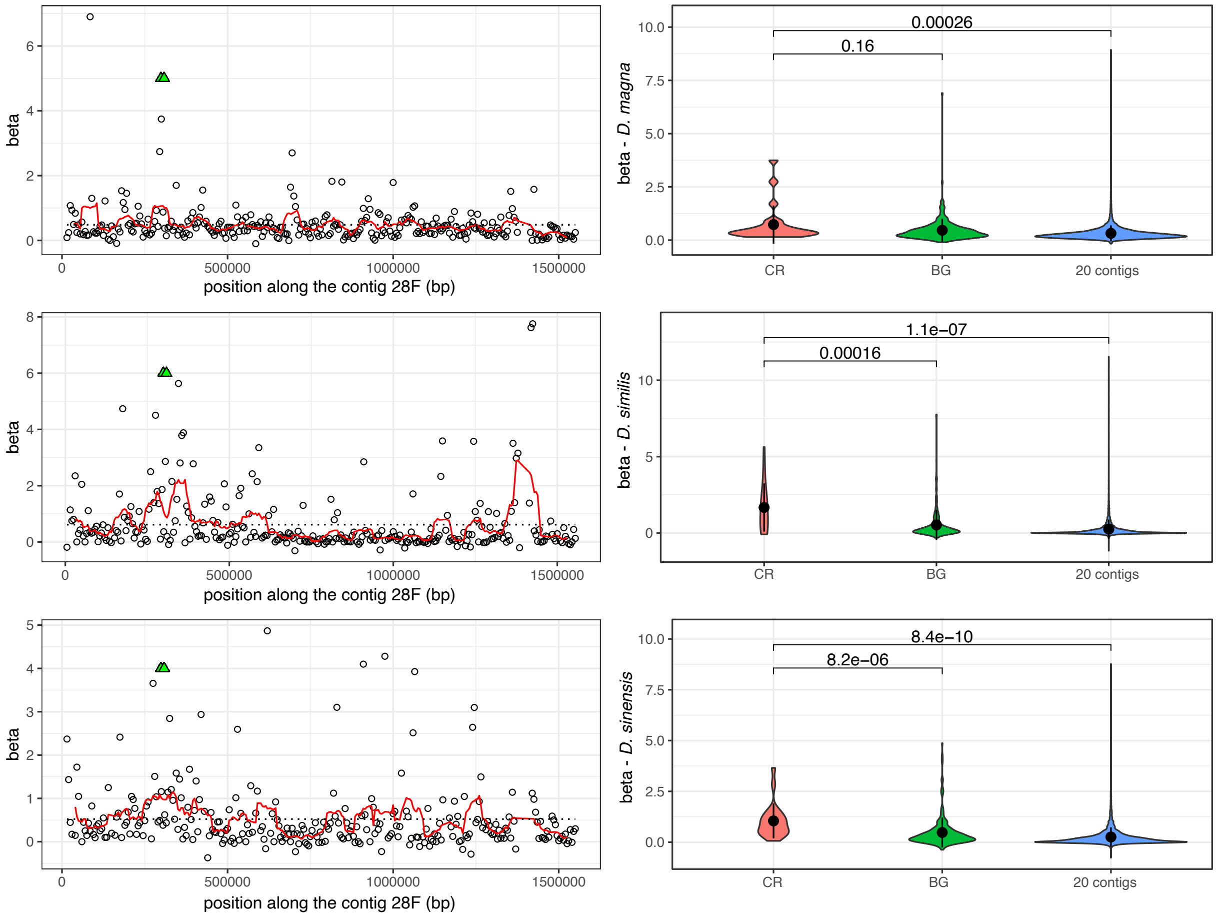

Supplementary Figure 19. Chromatogram of the amplicon 11F_1 of clone DE-S2-1 (*D. magna*)

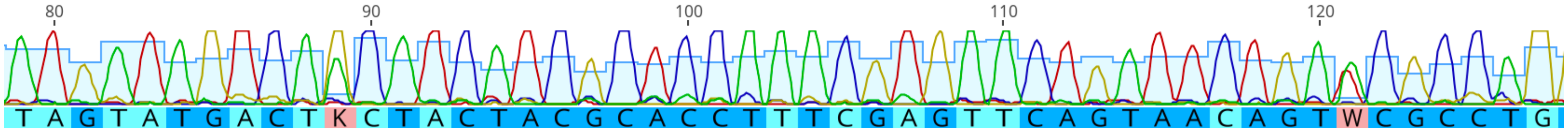

Supplementary Figure 20. Chromatogram of the amplicon 11F_2 of clone DE-S2-1 (*D. magna*)

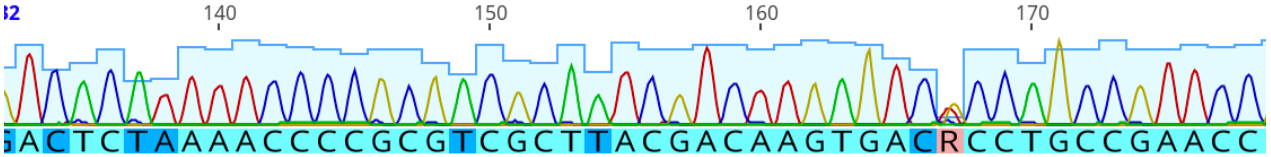

Supplementary Figure 21. Chromatogram of the amplicon 11F_2 of clone RU-NOV1-01 (*D. sinensis*)

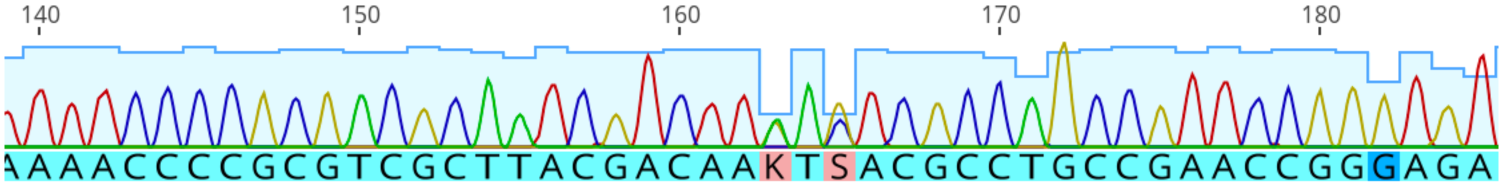

Supplementary Figure 22. Chromatogram of the amplicon 18F of clone RU-SDSP-82 (*D. sinensis*)

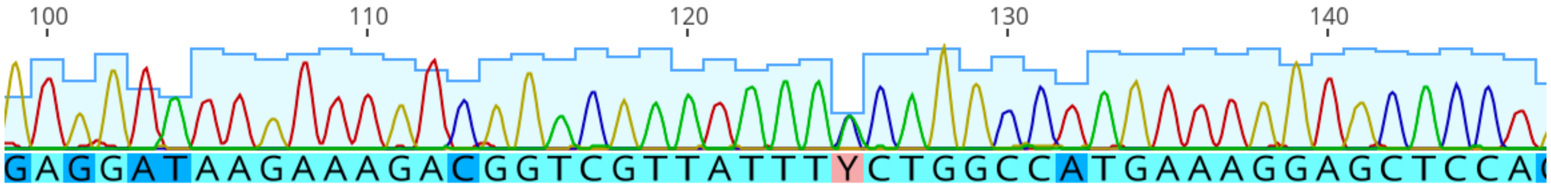

Supplementary Figure 23. Chromatogram of the amplicon 28F_1 of clone DE-S2-2 (*D. magna*)

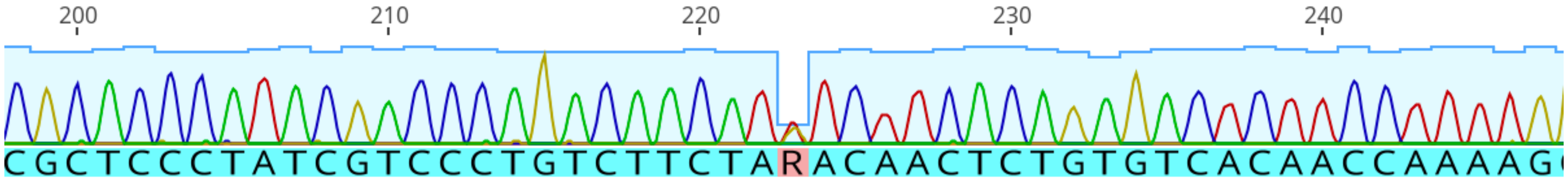

Supplementary Figure 24. Chromatogram of the amplicon 28F_1 of clone FR-TR-1 (*D. magna*)

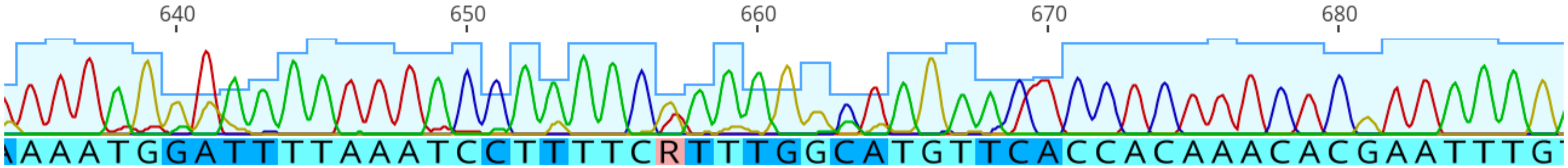

Supplementary Tables

Supplementary Table 1 | Summary of the number of SNPs observed in each of the five *Daphnia* lineages identified in this study.

Species	Lineage	Number of clones	number of SNPs
D. magna	Western Eurasian	126	1,210,667
	Eastern Asian	18	1,517,507
	North America	13	518,259
D. similis	-	15	539,092
D. sinensis	-	14	758,842

Supplementary Table 2. Resume of Wilcoxon rank sum tests that compared nucleotide diversity calculated in 5kb windows in the six candidate regions against the rest of the contig where the region lies and against the 20 longest contigs in *D. magna* genome not containing candidate regions. In bold P-values ≤ 0.05. Contig refers to the contig of *D. magna* reference genome 3.0 (Fields et al. in prep.). All tests are one-sided Wilcoxon tests.

Species	Contig	Region around TSP	vs rest of contig	vs 20 longest contigs
D. magna	000011F	2141058	***1.50E-05***	***1.70E-09***
	000011F	2343857	***0.0017***	***3.50E-06***
	000018F	1611159	0.096	***0.00028***
	000028F	298723	0.41	0.29
D. similis	000011F	2141056	***6.10E-04***	***1.10E-07***
	000011F	2343857	0.47	***0.0075***
	000018F	1611159	***0.0011***	***0.00026***
	000028F	298723	***1.60E-05***	***2.00E-06***
D. sinensis	000011F	2141056	***3.30E-05***	***4.90E-08***
	000011F	2343857	0.9	0.096
	000018F	1611159	***0.036***	***0.01***
	000028F	298723	***0.00063***	***7.70E-05***

Supplementary Table 3. Resume of Wilcoxon rank sum tests that compared nucleotide diversity calculated in 2.5kb windows in the six candidate regions against the rest of the contig where the region lies and against the 20 longest contigs in *D. magna* genome not containing candidate regions. In bold P-values ≤ 0.05. Contig refers to the contig of *D. magna* reference genome 3.0 (Fields et al. in prep.). All tests are one-sided Wilcoxon tests.

Species	Contig	Region around TSP	vs rest of contig	vs 20 longest contigs
D. magna	000011F	2141058	***1.20E-07***	***8.50E-14***
	000011F	2343857	***3.00E-07***	***2.30E-12***
	000018F	1611159	0.085	***4.30E-05***
	000028F	298723	0.55	***6.50E-02***
D. similis	000011F	2141056	***1.20E-02***	***1.80E-05***
	000011F	2343857	0.29	***0.0046***
	000018F	1611159	***5.50E-05***	***7.00E-06***
	000028F	298723	***2.50E-05***	***6.10E-07***
D. sinensis	000011F	2141056	***1.20E-04***	***1.80E-07***
	000011F	2343857	0.93	0.15
	000018F	1611159	***0.035***	***0.0084***
	000028F	298723	***0.00012***	***2.90E-06***

Supplementary Table 4. Resume of Wilcoxon rank sum tests that compared nucleotide diversity calculated in 1kb windows in the six candidate regions against the rest of the contig where the region lies and against the 20 longest contigs in *D. magna* genome not containing candidate regions. In bold P-values ≤ 0.05. Contig refers to the contig of *D. magna* reference genome 3.0 (Fields et al. in prep.). All tests are one-sided Wilcoxon tests.

Species	Contig	Region around TSP	vs rest of contig	vs 20 longest contigs
D. magna	000011F	2141058	***2.30E-15***	***2.22E-16***
	000011F	2343857	***4.20E-12***	***2.22E-16***
	000018F	1611159	***0.033***	***1.80E-07***
	000028F	298723	0.45	***1.70E-02***
D. similis	000011F	2141056	***4.60E-03***	***2.80E-06***
	000011F	2343857	0.69	0.33
	000018F	1611159	***5.80E-06***	***9.40E-08***
	000028F	298723	***2.30E-05***	***5.70E-08***
D. sinensis	000011F	2141056	***2.40E-05***	***1.60E-09***
	000011F	2343857	0.48	***0.032***
	000018F	1611159	***0.0011***	***1.10E-05***
	000028F	298723	***1.90E-05***	***2.70E-08***

Supplementary Table 5. Resume of Wilcoxon rank sum tests that compared Tajima's D calculated in 5kb windows in the six candidate regions against the rest of the contig where the region lies and against the 20 longest contigs in *D. magna* genome not containing candidate regions. In bold P-values ≤ 0.05. Contig refers to the contig of *D. magna* reference genome 3.0 (Fields et al. in prep.). All tests are one-sided Wilcoxon tests.

Species	Contig	Region around TSP	vs rest of contig	vs 20 longest contigs
D. magna	000011F	2141058	0.14	***6.20E-03***
	000011F	2343857	***0.02***	***8.00E-04***
	000018F	1611159	0.77	0.12
	000028F	298723	0.33	0.051
D. similis	000011F	2141056	***0.0073***	***0.00056***
	000011F	2343857	***0.00093***	***9.30E-05***
	000018F	1611159	0.16	0.06
	000028F	298723	***2.90E-05***	***4.40E-07***
D. sinensis	000011F	2141056	***1.80E-05***	***7.20E-07***
	000011F	2343857	***4.50E-05***	***1.90E-06***
	000018F	1611159	***0.03***	***0.0025***
	000028F	298723	***7.10E-05***	***7.90E-07***

Supplementary Table 6. Resume of Wilcoxon rank sum tests that compared Tajima's D calculated in 2.5kb windows in the six candidate regions against the rest of the contig where the region lies and against the 20 longest contigs in *D. magna* genome not containing candidate regions. In bold P-values ≤ 0.05. Contig refers to the contig of *D. magna* reference genome 3.0 (Fields et al. in prep.). All tests are one-sided Wilcoxon tests.

Species	Contig	Region around TSP	vs rest of contig	vs 20 longest contigs
D. magna	000011F	2141058	***5.30E-02***	***4.70E-04***
	000011F	2343857	***1.60E-03***	***1.00E-05***
	000018F	1611159	0.82	***7.10E-02***
	000028F	298723	0.47	***1.20E-01***
D. similis	000011F	2141056	***1.20E-01***	***1.10E-02***
	000011F	2343857	***0.0024***	***0.00015***
	000018F	1611159	1.80E-01	***4.20E-02***
	000028F	298723	***1.90E-05***	***3.50E-08***
D. sinensis	000011F	2141056	***2.50E-07***	***1.70E-09***
	000011F	2343857	***0.00048***	***2.50E-05***
	000018F	1611159	***0.059***	***0.0041***
	000028F	298723	***7.00E-07***	***9.80E-10***

Supplementary Table 7. Resume of Wilcoxon rank sum tests that compared Tajima's D calculated in 1kb windows in the six candidate regions against the rest of the contig where the region lies and against the 20 longest contigs in *D. magna* genome not containing candidate regions. In bold P-values ≤ 0.05. Contig refers to the contig of *D. magna* reference genome 3.0 (Fields et al. in prep.). All tests are one-sided Wilcoxon tests.

Species	Contig	Region around TSP	vs rest of contig	vs 20 longest contigs
D. magna	000011F	2141058	***2.80E-03***	***5.00E-07***
	000011F	2343857	***6.60E-05***	***5.80E-09***
	000018F	1611159	0.72	***2.90E-02***
	000028F	298723	0.51	***1.80E-02***
D. similis	000011F	2141056	***7.80E-02***	***3.20E-03***
	000011F	2343857	***2.20E-05***	***2.30E-07***
	000018F	1611159	***2.10E-02***	***1.80E-03***
	000028F	298723	***3.00E-08***	***4.90E-12***
D. sinensis	000011F	2141056	***4.40E-09***	***7.50E-12***
	000011F	2343857	***2.20E-06***	***5.50E-08***
	000018F	1611159	***0.0013***	***2.90E-05***
	000028F	298723	***2.10E-08***	***1.00E-11***

Supplementary Table 8. Resume of Wilcoxon rank sum tests that compared F_{st} calculated in 5kb windows in the six candidate regions against the rest of the contig where the region lies and against the 20 longest contigs in *D. magna* genome not containing candidate regions. In bold P-values ≤ 0.05. Contig refers to the contig of *D. magna* reference genome 3.0 (Fields et al. in prep.). All tests are one-sided Wilcoxon tests.

Species comparison	Contig	Region around TSP	vs rest of contig	vs 20 longest contig
D. magna vs *D. similis*	000011F	2141058	***6.20E-06***	***0.00011***
	000011F	2343857	***3.00E-08***	***6.30E-07***
	000018F	1611159	1	0.57
	000028F	298723	***0.05***	***0.0011***
D. similis vs *D. sinensis*	000011F	2141056	***0.0025***	0.069
	000011F	2343857	***3.70E-05***	***0.0023***
	000018F	1611159	0.078	***0.031***
	000028F	298723	***0.00065***	***1.60E-06***
D. magna vs *D. sinensis*	000011F	2141056	***2.00E-06***	***3.00E-05***
	000011F	2343857	***1.20E-08***	***3.10E-07***
	000018F	1611159	0.67	0.86
	000028F	298723	***0.048***	***0.0012***

Supplementary Table 9. Resume of Wilcoxon rank sum tests that compared F_{st} calculated in 2.5kb windows in the six candidate regions against the rest of the contig where the region lies and against the 20 longest contigs in *D. magna* genome not containing candidate regions. In bold P-values ≤ 0.05. Contig refers to the contig of *D. magna* reference genome 3.0 (Fields et al. in prep.). All tests are one-sided Wilcoxon tests.

Species comparison	Contig	Region around TSP	vs rest of contig	vs 20 longest contig
D. magna vs *D. similis*	000011F	2141058	***1.70E-08***	***2.10E-09***
	000011F	2343857	***7.70E-12***	***6.90E-12***
	000018F	1611159	0.68	0.23
	000028F	298723	0.055	***7.90E-05***
D. similis vs *D. sinensis*	000011F	2141056	***0.0038***	***0.001***
	000011F	2343857	***2.10E-05***	***9.50E-06***
	000018F	1611159	0.18	0.36
	000028F	298723	***2.90E-05***	***2.20E-09***
D. magna vs *D. sinensis*	000011F	2141056	***1.50E-09***	***3.40E-11***
	000011F	2343857	***2.20E-12***	***3.30E-13***
	000018F	1611159	0.8	0.16
	000028F	298723	***0.019***	***1.90E-05***

Supplementary Table 10. Resume of Wilcoxon rank sum tests that compared F_{st} calculated in 1kb windows in the six candidate regions against the rest of the contig where the region lies and against the 20 longest contigs in *D. magna* genome not containing candidate regions. In bold P-values ≤ 0.05. Contig refers to the contig of *D. magna* reference genome 3.0 (Fields et al. in prep.). All tests are one-sided Wilcoxon tests.

Species comparison	Contig	Region around TSP	vs rest of contig	vs 20 longest contig
D. magna vs *D. similis*	000011F	2141058	***1.80E-14***	***2.22E-16***
	000011F	2343857	***2.22E-16***	***2.22E-16***
	000018F	1611159	0.45	0.11
	000028F	298723	***0.0062***	***5.40E-09***
D. similis vs *D. sinensis*	000011F	2141056	***8.80E-04***	***2.00E-05***
	000011F	2343857	***8.20E-07***	***4.30E-08***
	000018F	1611159	***0.017***	0.57
	000028F	298723	***4.70E-07***	***1.50E-13***
D. magna vs *D. sinensis*	000011F	2141056	***3.80E-14***	***2.22E-16***
	000011F	2343857	***2.22E-16***	***2.22E-16***
	000018F	1611159	0.73	***0.039***
	000028F	298723	***0.0013***	***5.80E-10***

Supplementary Table 11. Resume of Wilcoxon rank sum tests that compared beta scores (-maf 0.05) calculated in 5kb windows in the six candidate regions against the rest of the contig where the region lies and against the 20 longest contigs in *D. magna* genome not containing candidate regions. In bold P-values ≤ 0.05. Contig refers to the contig of *D. magna* reference genome 3.0 (Fields et al. in prep.). All tests are one-sided Wilcoxon tests.

Species	Contig	Region around TSP	vs rest of contig	vs 20 longest contigs
D. magna	000011F	2141058	***0.002***	***8.80E-05***
	000011F	2343857	***9.30E-07***	***3.00E-08***
	000018F	1611159	0.91	0.28
	000028F	298723	0.16	***0.00026***
D. similis	000011F	2141056	***5.20E-05***	***2.30E-08***
	000011F	2343857	***0.00073***	***1.10E-05***
	000018F	1611159	0.23	***0.005***
	000028F	298723	***0.00016***	***1.10E-07***
D. sinensis	000011F	2141056	***0.0018***	***4.70E-06***
	000011F	2343857	0.1	***0.00048***
	000018F	1611159	0.22	***0.0028***
	000028F	298723	***8.20E-06***	***8.40E-10***

Supplementary Table 12. Resume of Wilcoxon rank sum tests that compared beta scores (-maf 0.1) calculated in 5kb windows in the six candidate regions against the rest of the contig where the region lies and against the 20 longest contigs in *D. magna* genome not containing candidate regions. In bold P-values ≤ 0.05. Contig refers to the contig of *D. magna* reference genome 3.0 (Fields et al. in prep.). All tests are one-sided Wilcoxon tests.

Species	Contig	Region around TSP	vs rest of contig	vs 20 longest contigs
D. magna	000011F	2141058	***1.50E-03***	***2.50E-05***
	000011F	2343857	***2.60E-06***	***3.40E-08***
	000018F	1611159	0.95	0.18
	000028F	298723	0.18	***0.0002***
D. similis	000011F	2141056	***3.40E-03***	***2.40E-06***
	000011F	2343857	***0.0013***	***3.50E-06***
	000018F	1611159	3.20E-01	***1.10E-02***
	000028F	298723	***1.30E-04***	***6.70E-08***
D. sinensis	000011F	2141056	***1.50E-03***	***2.30E-07***
	000011F	2343857	0.91	***1.50E-05***
	000018F	1611159	0.18	***0.00018***
	000028F	298723	***2.60E-05***	***3.50E-09***

Supplementary Table 13. Resume of Wilcoxon rank sum tests that compared beta scores (-maf 0.17) calculated in 5kb windows in the six candidate regions against the rest of the contig where the region lies and against the 20 longest contigs in *D. magna* genome not containing candidate regions. In bold P-values ≤ 0.05. Contig refers to the contig of *D. magna* reference genome 3.0 (Fields et al. in prep.). All tests are one-sided Wilcoxon tests.

Species	Contig	Region around TSP	vs rest of contig	vs 20 longest contigs
D. magna	000011F	2141058	***2.60E-03***	***4.90E-05***
	000011F	2343857	***1.10E-05***	***1.30E-07***
	000018F	1611159	0.79	0.33
	000028F	298723	0.29	***0.00076***
D. similis	000011F	2141056	***6.90E-03***	***1.40E-05***
	000011F	2343857	***0.013***	***8.40E-05***
	000018F	1611159	3.00E-01	***2.30E-02***
	000028F	298723	***1.10E-04***	***1.90E-07***
D. sinensis	000011F	2141056	***8.60E-04***	***2.70E-08***
	000011F	2343857	0.17	***2.40E-04***
	000018F	1611159	0.097	***4.50E-05***
	000028F	298723	***2.60E-06***	***3.40E-10***

Supplementary Table 14. Resume of Wilcoxon rank sum tests that compared beta scores (-maf 0.05) calculated in 2.5kb windows in the six candidate regions against the rest of the contig where the region lies and against the 20 longest contigs in *D. magna* genome not containing candidate regions. In bold P-values ≤ 0.05. Contig refers to the contig of *D. magna* reference genome 3.0 (Fields et al. in prep.). All tests are one-sided Wilcoxon tests.

Species	Contig	Region around TSP	vs rest of contig	vs 20 longest contigs
D. magna	000011F	2141058	***2.20E-04***	***1.30E-05***
	000011F	2343857	***2.80E-10***	***3.20E-12***
	000018F	1611159	0.92	0.63
	000028F	298723	0.32	***4.20E-03***
D. similis	000011F	2141056	***1.60E-05***	***8.00E-09***
	000011F	2343857	***0.0016***	***6.60E-05***
	000018F	1611159	***3.70E-02***	***1.20E-03***
	000028F	298723	***2.30E-04***	***1.00E-07***
D. sinensis	000011F	2141056	***9.90E-03***	***3.30E-05***
	000011F	2343857	0.08	***0.003***
	000018F	1611159	0.52	***2.00E-02***
	000028F	298723	***2.40E-05***	***2.40E-09***

Supplementary Table 15. Resume of Wilcoxon rank sum tests that compared beta scores (-maf 0.1) calculated in 2.5kb windows in the six candidate regions against the rest of the contig where the region lies and against the 20 longest contigs in *D. magna* genome not containing candidate regions. In bold P-values ≤ 0.05. Contig refers to the contig of *D. magna* reference genome 3.0 (Fields et al. in prep.). All tests are one-sided Wilcoxon tests.

Species	Contig	Region around TSP	vs rest of contig	vs 20 longest contigs
D. magna	000011F	2141058	***1.30E-04***	***1.50E-07***
	000011F	2343857	***1.80E-09***	***1.40E-12***
	000018F	1611159	0.73	0.063
	000028F	298723	0.24	***0.00014***
D. similis	000011F	2141056	***7.30E-03***	***3.50E-06***
	000011F	2343857	***0.0023***	***7.10E-06***
	000018F	1611159	1.20E-01	***2.90E-04***
	000028F	298723	***2.30E-04***	***5.20E-09***
D. sinensis	000011F	2141056	***7.40E-03***	***9.80E-08***
	000011F	2343857	0.88	***3.80E-05***
	000018F	1611159	0.44	***4.10E-05***
	000028F	298723	***1.70E-05***	***1.20E-11***

Supplementary Table 16. Resume of Wilcoxon rank sum tests that compared beta scores (-maf 0.17) calculated in 2.5kb windows in the six candidate regions against the rest of the contig where the region lies and against the 20 longest contigs in *D. magna* genome not containing candidate regions. In bold P-values ≤ 0.05. Contig refers to the contig of *D. magna* reference genome 3.0 (Fields et al. in prep.). All tests are one-sided Wilcoxon tests.

Species	Contig	Region around TSP	vs rest of contig	vs 20 longest contigs
D. magna	000011F	2141058	***6.40E-04***	***8.50E-07***
	000011F	2343857	***7.40E-08***	***5.80E-11***
	000018F	1611159	0.91	0.12
	000028F	298723	0.44	***0.001***
D. similis	000011F	2141056	***2.50E-02***	***2.30E-04***
	000011F	2343857	***0.027***	***1.70E-03***
	000018F	1611159	1.40E-01	***2.00E-03***
	000028F	298723	***1.00E-04***	***3.70E-09***
D. sinensis	000011F	2141056	***1.40E-03***	***2.80E-08***
	000011F	2343857	0.25	***1.50E-03***
	000018F	1611159	0.28	***8.60E-05***
	000028F	298723	***9.40E-06***	***5.90E-11***

Supplementary Table 17. Resume of Wilcoxon rank sum tests that compared beta scores (-maf 0.05) calculated in 1kb windows in the six candidate regions against the rest of the contig where the region lies and against the 20 longest contigs in *D. magna* genome not containing candidate regions. In bold P-values ≤ 0.05. Contig refers to the contig of *D. magna* reference genome 3.0 (Fields et al. in prep.). All tests are one-sided Wilcoxon tests.

Species	Contig	Region around TSP	vs rest of contig	vs 20 longest contigs
D. magna	000011F	2141058	***3.30E-07***	***6.40E-06***
	000011F	2343857	***5.20E-16***	***2.22E-16***
	000018F	1611159	0.77	0.081
	000028F	298723	0.14	0.077
D. similis	000011F	2141056	***1.60E-04***	***5.30E-05***
	000011F	2343857	***0.0037***	***5.30E-03***
	000018F	1611159	***1.50E-03***	***1.20E-03***
	000028F	298723	***1.70E-06***	***2.50E-08***
D. sinensis	000011F	2141056	***1.00E-02***	***1.40E-03***
	000011F	2343857	0.082	***0.021***
	000018F	1611159	0.16	0.054
	000028F	298723	***1.30E-08***	***2.30E-13***

Supplementary Table 18. Resume of Wilcoxon rank sum tests that compared beta scores (-maf 0.1) calculated in 1kb windows in the six candidate regions against the rest of the contig where the region lies and against the 20 longest contigs in *D. magna* genome not containing candidate regions. In bold P-values ≤ 0.05. Contig refers to the contig of *D. magna* reference genome 3.0 (Fields et al. in prep.). All tests are one-sided Wilcoxon tests.

Species	Contig	Region around TSP	vs rest of contig	vs 20 longest contigs
D. magna	000011F	2141058	***1.60E-07***	***7.60E-13***
	000011F	2343857	***2.50E-15***	***2.22E-16***
	000018F	1611159	0.83	0.098
	000028F	298723	0.11	***3.80E-06***
D. similis	000011F	2141056	***2.00E-03***	***1.30E-07***
	000011F	2343857	***0.0057***	***1.90E-05***
	000018F	1611159	7.60E-02	***1.20E-04***
	000028F	298723	***3.10E-07***	***1.10E-15***
D. sinensis	000011F	2141056	***4.20E-03***	***6.70E-10***
	000011F	2343857	0.094	***1.70E-07***
	000018F	1611159	0.22	***5.20E-07***
	000028F	298723	***5.90E-09***	***2.22E-16***

Supplementary Table 19. Resume of Wilcoxon rank sum tests that compared beta scores (-maf 0.17) calculated in 1kb windows in the six candidate regions against the rest of the contig where the region lies and against the 20 longest contigs in *D. magna* genome not containing candidate regions. In bold P-values ≤ 0.05. Contig refers to the contig of *D. magna* reference genome 3.0 (Fields et al. in prep.). All tests are one-sided Wilcoxon tests.

Species	Contig	Region around TSP	vs rest of contig	vs 20 longest contigs
D. magna	000011F	2141058	***5.20E-06***	***1.60E-10***
	000011F	2343857	***6.50E-13***	***2.22E-16***
	000018F	1611159	0.99	0.13
	000028F	298723	0.2	***7.70E-05***
D. similis	000011F	2141056	5.50E-02	***2.60E-03***
	000011F	2343857	***0.032***	***3.30E-03***
	000018F	1611159	4.30E-01	5.70E-02
	000028F	298723	***8.20E-08***	***1.60E-15***
D. sinensis	000011F	2141056	***8.10E-04***	***1.40E-09***
	000011F	2343857	0.071	***1.80E-06***
	000018F	1611159	***0.029***	***3.70E-07***
	000028F	298723	***4.40E-08***	***2.40E-15***

Supplementary Table 20. Primers and PCR conditions used for the amplification of the amplicons surrounding the TSPs

Amplicon	TSP position (bp on contig)	Primer F	Primer R	Annealing temperature (°C)
11F_1	2141058	GCATCGTCAGGGAGTTTGGA	GCAGCAGGCAAATCAAAGCT	57
11F_2	2343857	CGTTTGCCAATGGTCCGTTT	ACCGAGTTTTCTCCCGGTTC	56
18F	1611159	CGTTAATGGCGACGCTTCTG	CATTCCACCCAGCTCCATGT	56
28F_1	298723	GCGCCAAACCCTCAACAATT	TTCCAACCAGATCACCCACG	60
