# Supplementary material for: Long-term balancing selection for pathogen resistance maintains trans-species polymorphisms in a planktonic crustacean
Source: Nat Commun. 2024 Jun 22;15:5333. doi: 10.1038/s41467-024-49726-8 (PMC11193740; doi:10.1038/s41467-024-49726-8)
Supplement: Supplementary file 3 — Description of Additional Supplementary Files [file 41467_2024_49726_MOESM3_ESM.pdf]

Description of Additional Supplementary Materials for

Long-term balancing selection for pathogen resistance maintains trans-species polymorphisms in a planktonic crustacean

Luca Cornetti[1,+], Peter D. Fields[1], Louis Du Pasquier[1] & Dieter Ebert[1] ✉

[1] *University of Basel, Department of Environmental Sciences, Zoology, Basel, Switzerland*

✉ email: dieter.ebert@unibas.ch

+ current address: Syngenta Crop Protection AG, CH-4332 Stein, Switzerland

Legends for each Supplementary Data file.

Supplementary Data 1 | Summary of the 186 *Daphnia* clones included in this study. Location details are reported (Country, Latitude and Longitude) as well as their resistotypes relative to different *Pasteuria* strains. We assessed whether labelled spores attached to the host's foregut (all five *Pasteuria* strains) or/and hindgut (two *Pasteuria* strains: P15 and P21); R= resistant; S= susceptible.

Supplementary Data 2| Summary of resistance assessment (attachment test) and infection trials in *D. similis* and *D. sinensis*.
The columns including R and S indicate whether the genotype is resistant or susceptible, respectively, to the five *Pasteuria* strains. Genotypes that resulted susceptible to the attachment test (at least in one of the two attachment sites for P15 and P21) were used for the infection trials that consisted of five or ten replicates per genotype. Genotypes showing the signature of infection in at least one of the replicates are considered infectable and indicated with I. Genotype highlighted with NI (Not Infectable) never showed presence of *Pasteuria* spores, suggesting that the bacteria can attach but do not successfully replicate inside the host's body.

Supplementary Data 3 | List of shared polymorphisms and their minor allele frequency within each of the five lineages.
Contig refers to the contig of *D. magna* reference genome 3.0 (Fields et al. in prep.). *D. magna* WE is the Western Eurasian clade of this species, EA is the East Asian clade and NA the North American clade.

Supplementary Data 4 | Table summarizing, for each of the local allele trees built in the surrounding of the 131 SNPs retained for this analysis, the probability of having haplotypes clustering by allele ($P_{allelic}$). This is reported for trees built with window size around the focal SNP of 100 to 1500 basepair (bp) length. The focal SNP was excluded from the sequence before tree building. The reported probability is the proportion of trees having haplotypes clustering by allele over the 1000 resampled trees. Colors are proportional to the observed $P_{allelic}$, with red showing the highest probability (100%) and green showing the lowest probability (0%). Contig refers to the contig of *D. magna* reference genome 3.0 (Fields et al. in prep.).

Supplementary Data 5 | Annotation and predicted effect of the 56 shared SNPs with evidence of being, or being in LD with, a TSP, i.e. these 56 shared SNPs occurred within the 11 regions with at least two shared SNPs in LD. Each of these 11 regions has at least one putative TSP identified (in bold these SNPs with a $P_{allelic}$ higher than 85). Contig refers to the contig of *D. magna* reference genome 3.0 (Fields et al. in prep.).

Supplementary Data 6 | Details of the 11 genes that included at least one putative TSP. PtoD is corrected for gene length and its value multiplied by 1000. In **bold** the four genes that showed exceptional variability with their average percentile (last column) falling in the top 10 % of the PtoD distribution. Asterisks indicate gene whose functional annotation was obtained after an in-depth investigation. Contig refers to the contig of *D. magna* reference genome 3.0 (Fields et al. in prep.).
