# Supplementary material for: Long-term balancing selection for pathogen resistance maintains trans-species polymorphisms in a planktonic crustacean
Source: Nat Commun. 2024 Jun 22;15:5333. doi: 10.1038/s41467-024-49726-8 (PMC11193740; doi:10.1038/s41467-024-49726-8)
Supplement: Supplementary file 5 — Reporting Summary [file 41467_2024_49726_MOESM5_ESM.pdf]

nature portfolio

Corresponding author(s): Dieter Ebert

Last updated by author(s): May 5, 2024

Reporting Summary

Nature Portfolio wishes to improve the reproducibility of the work that we publish. This form provides structure for consistency and transparency in reporting. For further information on Nature Portfolio policies, see our Editorial Policies and the Editorial Policy Checklist.

Statistics

For all statistical analyses, confirm that the following items are present in the figure legend, table legend, main text, or Methods section.

n/a	Confirmed	
☐	☒	The exact sample size (*n*) for each experimental group/condition, given as a discrete number and unit of measurement
☐	☒	A statement on whether measurements were taken from distinct samples or whether the same sample was measured repeatedly
☐	☒	The statistical test(s) used AND whether they are one- or two-sided<br>*Only common tests should be described solely by name; describe more complex techniques in the Methods section.*
☒	☐	A description of all covariates tested
☒	☐	A description of any assumptions or corrections, such as tests of normality and adjustment for multiple comparisons
☐	☒	A full description of the statistical parameters including central tendency (e.g. means) or other basic estimates (e.g. regression coefficient) AND variation (e.g. standard deviation) or associated estimates of uncertainty (e.g. confidence intervals)
☐	☒	For null hypothesis testing, the test statistic (e.g. *F*, *t*, *r*) with confidence intervals, effect sizes, degrees of freedom and *P* value noted<br>*Give P values as exact values whenever suitable.*
☒	☐	For Bayesian analysis, information on the choice of priors and Markov chain Monte Carlo settings
☒	☐	For hierarchical and complex designs, identification of the appropriate level for tests and full reporting of outcomes
☒	☐	Estimates of effect sizes (e.g. Cohen's *d*, Pearson's *r*), indicating how they were calculated

Our web collection on statistics for biologists contains articles on many of the points above.

Software and code

Policy information about availability of computer code

Data collection	no software was used to collect data
Data analysis	No software, not yet described in published literature, was used to analyze data. Scripts required for replicating our analysis are available at GitHub: https://github.com/ebertlab/transpecies_polymorphism_manuscript.

For manuscripts utilizing custom algorithms or software that are central to the research but not yet described in published literature, software must be made available to editors and reviewers. We strongly encourage code deposition in a community repository (e.g. GitHub). See the Nature Portfolio guidelines for submitting code & software for further information.

Data

Policy information about availability of data

All manuscripts must include a data availability statement. This statement should provide the following information, where applicable:

- Accession codes, unique identifiers, or web links for publicly available datasets
- A description of any restrictions on data availability
- For clinical datasets or third party data, please ensure that the statement adheres to our policy

All newly generated genomic data will be available from NCBI under the BioProjectID PRJNA995356. The utilized VCF is available at Zenodo under the following link: https://zenodo.org/records/8153123

Research involving human participants, their data, or biological material

Policy information about studies with human participants or human data. See also policy information about sex, gender (identity/presentation), and sexual orientation and race, ethnicity and racism.

Reporting on sex and gender	n/a
Reporting on race, ethnicity, or other socially relevant groupings	n/a
Population characteristics	n/a
Recruitment	n/a
Ethics oversight	n/a

Note that full information on the approval of the study protocol must also be provided in the manuscript.

Field-specific reporting

Please select the one below that is the best fit for your research. If you are not sure, read the appropriate sections before making your selection.

☐ Life sciences ☐ Behavioural & social sciences ☒ Ecological, evolutionary & environmental sciences

For a reference copy of the document with all sections, see nature.com/documents/nr-reporting-summary-flat.pdf

Ecological, evolutionary & environmental sciences study design

All studies must disclose on these points even when the disclosure is negative.

Study description	Whole genome analyses to look for evidence of long-term balancing selection.
Research sample	186 Daphnia genotypes were sequenced. These include samples of three Daphnia species, collected across the site on the Nortthern Hemisphere to cover the known species' distribution. We included all populations we could find. No populations were excluded, unless several populations where very close to each other (to avoid pseudo-replication).
Sampling strategy	For this study one genotype per sampling location was analyzed. We included all populations we could find. No populations were excluded, unless several populations where very close to each other (to avoid pseudo-replication).
Data collection	Different collaborators helped to collect Daphnia genotypes. They recorded GPS coordinates of the sampling location using hand-held GPS devices or high resolution maps.
Timing and spatial scale	We collected Daphnia magna genotypes from the entire Holarctic and much of Eurasia for the D. similis and D. sinensis between 03/1994 and 11/2014. These samples form the Daphnia diversity panel, a panel that was accumulated across many years.
Data exclusions	No data were excluded from the analyses
Reproducibility	The infection experiments were repeated multiple times to verify reproducibility
Randomization	This does not apply because this is not an experimental study.
Blinding	This does not apply, because this is not an experimental study.

Did the study involve field work? ☐ Yes ☒ No

Reporting for specific materials, systems and methods

We require information from authors about some types of materials, experimental systems and methods used in many studies. Here, indicate whether each material, system or method listed is relevant to your study. If you are not sure if a list item applies to your research, read the appropriate section before selecting a response.

Materials & experimental systems

n/a	Involved in the study
☒	☐ Antibodies
☒	☐ Eukaryotic cell lines
☒	☐ Palaeontology and archaeology
☐	☒ Animals and other organisms
☒	☐ Clinical data
☒	☐ Dual use research of concern
☒	☐ Plants

Methods

n/a	Involved in the study
☒	☐ ChIP-seq
☒	☐ Flow cytometry
☒	☐ MRI-based neuroimaging

Animals and other research organisms

Policy information about studies involving animals; ARRIVE guidelines recommended for reporting animal research, and Sex and Gender in Research

Laboratory animals	The study did not involve laboratory animals
Wild animals	The study concerns only lower invertebrates (planktonic crustaceans), which were originally collected from natural population, and cloned in the laboratory. Only females were collected. They were kept by clonal propagation with about 15 to 20 generations per year in the laboratory. They are part of a collection on lines called "The Daphnia Diversity Panel". In no case did we sequence animals directly collected from the field.
Reporting on sex	All animals sequenced were females. Water fleas reproduce asexual and therefore males are rare.
Field-collected samples	Each genotype was collected from a different population and was kept clonally (iso-female lines) in the laboratory in standard culture conditions: 20°C, a light : dark cycle of 16 : 8, artificial Daphnia medium (ADaM), and a 1 : 1 (by cell count) mixture of Nanochloropsis limnetica and Tetradesmus obliquus algae as the only food (50 million cells per 380 ml jar three times a week). These iso-females lines of water fleas have been kept for many years in lab-cultures.In no case have been animals used to collect data or DNA that were collected from the field. All animals used in the study have been breed in the laboratory.
Ethics oversight	No ethical approval or guidance was required, because collecting and working with zooplankton does not require ethical approval. Approving experiments in not required (not even recommend) for water fleas.

Note that full information on the approval of the study protocol must also be provided in the manuscript.
